# Characterization of Cellular Heterogeneity and an Immune Subpopulation of Human Megakaryocytes

**DOI:** 10.1002/advs.202100921

**Published:** 2021-05-26

**Authors:** Cuicui Liu, Dan Wu, Meijuan Xia, Minmin Li, Zhiqiang Sun, Biao Shen, Yiying Liu, Erlie Jiang, Hongtao Wang, Pei Su, Lihong Shi, Zhijian Xiao, Xiaofan Zhu, Wen Zhou, Qianfei Wang, Xin Gao, Tao Cheng, Jiaxi Zhou

**Affiliations:** ^1^ State Key Laboratory of Experimental Hematology National Clinical Research Center for Blood Diseases Institute of Hematology and Blood Diseases Hospital Chinese Academy of Medical Sciences and Peking Union Medical College Tianjin 300020 China; ^2^ Center for Stem Cell Medicine Chinese Academy of Medical Sciences and Department of Stem Cells and Regenerative Medicine Peking Union Medical College Tianjin 300020 China; ^3^ Key Laboratory of Carcinogenesis and Cancer Invasion Ministry of Education Key Laboratory of Carcinogenesis National Health and Family Planning Commission Cancer Research Institute School of Basic Medical Science Central South University Changsha 410078 China; ^4^ Key Laboratory of Genomic and Precision Medicine Collaborative Innovation Center of Genetics and Development Beijing Institute of Genomics Chinese Academy of Sciences Beijing 100101 China

**Keywords:** CD48, cellular heterogeneity, human megakaryopoiesis, immune‐surveillance, single‐cell RNA‐Seq

## Abstract

Megakaryocytes (MKs) and their progeny platelets function in a variety of biological processes including coagulation, hemostasis, inflammation, angiogenesis, and innate immunity. However, the divergent developmental and cellular landscape of adult MKs remains mysterious. Here, by deriving the single‐cell transcriptomic profiling of MKs from human adult bone marrow (BM), cellular heterogeneity within MKs is unveiled and an MK subpopulation with high enrichment of immune‐associated genes is identified. By performing the dynamic single‐cell transcriptomic landscape of human megakaryopoiesis in vitro, it is found that the immune signatures of MKs can be traced back to the progenitor stage. Furthermore, two surface markers, CD148 and CD48, are identified for mature MKs with immune characteristics. At the functional level, these CD148^+^CD48^+^ MKs can respond rapidly to immune stimuli both in vitro and in vivo, exhibit high‐level expression of immune receptors and mediators, and may function as immune‐surveillance cells. The findings uncover the cellular heterogeneity and a novel immune subset of human adult MKs and should greatly facilitate the understanding of the divergent functions of MKs under physiological and pathological conditions.

## Graphical abstract

By performing single‐cell transcriptomic analysis of human megakaryocytes from native bone marrow, the megakaryocyte heterogeneity is revealed, an immune subpopulation marked by CD148 and CD48 is discovered, and the potential function of immune megakaryocytes as immune‐surveillance cells is elucidated.

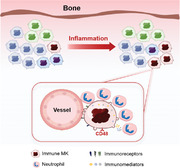

## 1 Introduction

Megakaryocytes (MKs) are large (50–100 µm) and rare (≈0.05%) hematopoietic cells enriched in the bone marrow (BM) and have well‐established functions in platelet production.^[^ ^1^ ^]^ It is traditionally thought that MKs arise from hematopoietic stem cells (HSCs) in the BM via common myeloid progenitors (CMPs), megakaryocyte‐erythroid progenitors (MEPs), and, subsequently, the MK progenitors (MKPs), which undergo a unique maturation process including endomitosis and cytoplasmic expansion, leading ultimately to the generation of terminal MKs and platelets.^[^ ^2^ ^]^ Recent studies have begun to redefine this hierarchy and shed new light on alternative routes from MK‐biased HSCs,^[^ ^3^, ^4^, ^5^ ^]^ multipotential progenitor population (MPP2)^[^ ^6^ ^]^ or CD41^+^ CMPs,^[^ ^7^ ^]^ independent of the canonical megakaryocyte‐erythroid lineage bifurcation.

Emerging evidence has demonstrated that MKs are essential for many other pathophysiological processes beyond platelet production, such as HSC quiescence, inflammation, immunity, and bone metastasis.^[^ ^8^, ^9^ ^]^ For example, MKs can produce the extracellular matrix to help establish the BM niches.^[^ ^10^ ^]^ As HSC‐derived niche cells, MKs maintain homeostatic quiescence through the production of PF4 and TGF*β*1 and promote regeneration of HSCs post‐injury via FGF1 secretion.^[^ ^11^ ^]^ In addition, an increase in MKs occurs in response to the entrance of metastatic cells to the BM, although the roles of MKs in modulating bone metastasis of different tumor types are still being debated.^[^ ^9^ ^]^ More importantly, MKs are found to express surface molecules related to inflammation or adaptive immunity and appear to have the potential function as immune cells.^[^ ^8^ ^]^ Indeed, recent studies have begun to unveil some intriguing details underlying the potential immune functions of MKs. For example, the MKs in the mouse lung express higher levels of immune molecules compared to BM MKs, playing the key immune regulatory roles.^[^ ^12^ ^]^ Furthermore, mature MKs with the expression of MHC class I, which function as potential novel antigen‐presenting cells, can process and present endogenous/exogenous antigens on MHC class I molecules to activate antigen‐specific CD8^+^ T cells.^[^ ^13^ ^]^ Moreover, MKs can transfer MHC class I molecules loaded with foreign antigens to platelets, potentially enabling and amplifying the antigen cross‐presentation.^[^ ^13^ ^]^ In addition, MKs can secrete multiple mediators to affect other immune cells via binding to their surface receptors.^[^ ^8^ ^]^

Despite the accumulating evidence for the diverse functions of MKs, outstanding questions remain unanswered. Specifically, do MKs fulfill these distinct functions by using a rather homogeneous MK population or distinct heterogeneous subpopulations with devoted responsibilities? Furthermore, if MK heterogeneity does exist, how does human megakaryopoiesis take place to result in such heterogeneity?

Recently, we performed the single‐cell transcriptomic profiling of MKs from human yolk sac and fetal liver, decoding the cellular heterogeneity and developmental trajectories of early megakaryopoiesis for the first time.^[^ ^14^ ^]^ However, due to the technical limitation of MK isolation from the native BM, the potential heterogeneity of human adult MKs remains relatively understudied at a single‐cell resolution. In this study, we successfully isolated and enriched the mature MKs from human adult BM using an improved efficient isolation strategy and uncovered the transcriptomic and cellular heterogeneity of adult MKs for the first time. We also constructed a comprehensive molecular landscape of human megakaryopoiesis in vitro. A distinct MK subpopulation with strong immune characteristics were identified consistently both in vivo and in vitro. Remarkably, these immune MKs can be identified via the coexpression of CD48 and CD148, a surface marker highly specific for mature MKs, and are significantly elevated when exposed to immune stimuli. Furthermore, CD148^+^CD48^+^ MKs, which express high‐level immune receptors and mediators, might act as potential immunosensors during acute inflammation. Our findings provide new insights into the poorly defined cellular heterogeneity of human adult MKs and should facilitate the exploration of the diverse functions of MKs under various physiological and pathological conditions.

## 2 Results

### 2.1 Single‐Cell Profiling of Human MKs from Native Bone Marrow

**Figure 1:**
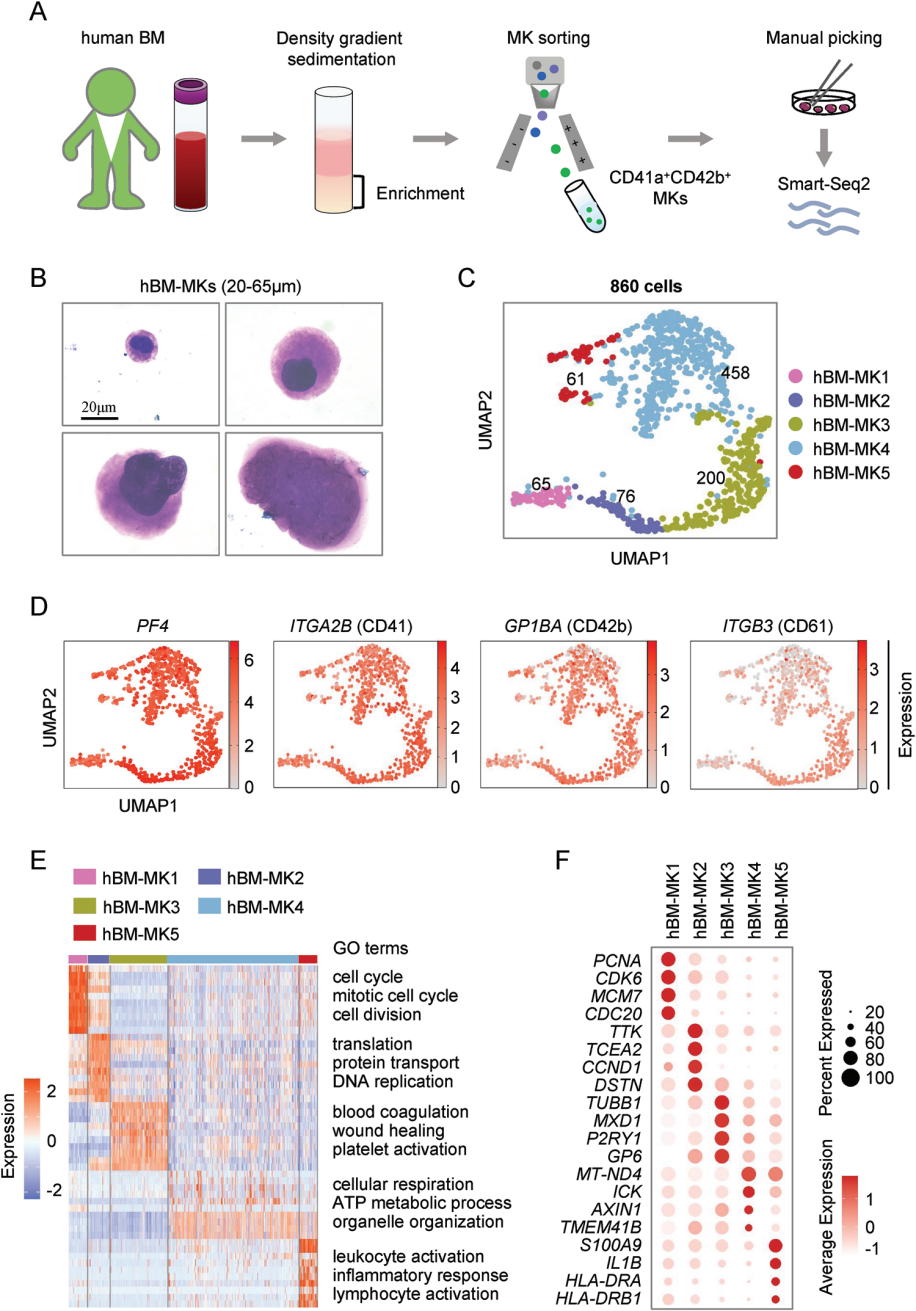
Single‐cell profiling of human MKs from native bone marrow. A) Schematic diagram of single‐cell RNA‐seq of human MKs from native bone marrow (BM). B) Representative morphologies of MKs with different sizes analyzed by MGG staining (scale bar, 20 µm). C) Cell clusters of 860 human MK single cells visualized by UMAP. Colors indicate different MK clusters. Each dot represents one cell. D) UMAP visualization of the expression of known marker genes for MK clusters. E) Heatmap showing top ten highly differentially expressed genes in each cluster with gene ontology (GO) analysis. Highlighted GO terms were selected by adjusted *P* value (<0.05). F) Dot plot showing the expression of feature genes in five distinct MK clusters.

MKs function in a variety of biological processes including coagulation, hemostasis, inflammation, angiogenesis, and innate immunity.^[^ ^8^ ^]^ However, whether the diverse functions of MKs are fulfilled by the entire MK population as a whole or distinct subpopulations of MKs with dedicated functions remains unknown. This is partially attributed to the technical challenge to isolate intact MKs from human bone marrow (hBM) because of their large size and fragility. To characterize potential cellular heterogeneity using MKs from hBM, we modified the previous described method,^[^ ^15^ ^]^ allowing us to successfully isolate intact MKs from hBM (**Figure** **1**A). The CD41a^+^CD42b^+^ MKs were sorted from the BM suspension and showed intact structure with various sizes (Figure S1A,B, Supporting Information). Further ploidy analysis showed that the MKs ranged from 2N to 64N, while around 60% of them exhibited more than 16N, indicating that our method produced mature MKs with higher ploidy (Figure S1C, Supporting Information). These MKs also showed various morphologies, as revealed by May–Grünwald–Giemsa (MGG) staining (Figure 1B).

We next manually collected ≈1,100 MKs under the microscope and applied these MKs to Smart‐Seq2 sequencing by following standard protocols (Figure 1A). These cells were collected from the BM of 15–48 year old donors, and the number of MKs collected from each donation was listed (Figure S1D, Supporting Information). We obtained, for the first time, the single‐cell transcriptomic profiling of adult MKs from hBM (Figure 1C).

After quality control, 860 qualified MKs, visualized by using uniform manifold approximation and projection (UMAP), were divided into 5 subpopulations, termed as hBM‐MK1 to hBM‐MK5, respectively (Figure 1C), and each subpopulation was present in different age cohorts (Figure S1E,F, Supporting Information). Among them, *PF4* (platelet factor 4), *ITGA2B* (CD41), *GP1BA* (CD42b), and *ITGB3* (CD61), well‐established markers for MKs, were highly expressed in these subpopulations (Figure 1D; Figure S1G, Supporting Information), indicating that our isolation methods were reliable and produced only MKs.

To explore potential cellular heterogeneity of collected MKs, we next conducted gene ontogeny (GO) analysis, which showed that distinct gene sets were enriched in a specific subpopulation of MKs (Figure 1E). Specially, cell cycle, cell division, and mitosis‐associated genes, such as *PCNA* (proliferating cell nuclear antigen), *CDK6* (cyclin dependent kinase 6), *MCM7* (minichromosome maintenance complex component 7), and *CDC20* (cell division cycle 20), were enriched in hBM‐MK1, indicating that they are MK progenitor cells undergoing active cell proliferation (Figure 1E,F; Figure S1H, Supporting Information). hBM‐MK2 was mainly enriched in DNA replication and protein translation or transport‐associated genes, such as *TTK* (TTK protein kinase), *TCEA2* (transcription elongation factor A2), and *DSTN* (destrin, actin depolymerizing factor) (Figure 1E,F). The genes related to MK polyploidization (Table S1, Supporting Information), including *CCND1* (cyclin D1), which play a key role in the endomitotic cell cycle,^[^ ^16^ ^]^ were also highly expressed in hBM‐MK2 (Figure 1F; Figure S1H, Supporting Information), thus indicating that hBM‐MK2 consisted of cells undergoing the polyploidization process. The GO analysis revealed the enrichment of genes related to blood coagulation, wound healing and platelet activation in hBM‐MK3 (Figure 1E). For instance, genes associated with platelet function, including *GP6* (glycoprotein 6), a collagen receptor important for collagen‐induced platelet aggregation and thrombus formation,^[^ ^17^ ^]^ and *P2RY1* (purinergic receptor P2Y1), an ATP receptor selectively regulating platelet aggregation,^[^ ^18^ ^]^ were found in hBM‐MK3 (Figure 1F). In addition, genes important for thrombopoiesis, such as MK‐specific skeleton protein *TUBB1* (tubulin beta 1 Class VI), and transcription factor (TF) *MXD1* (MAX dimerization protein 1),^[^ ^19^, ^20^ ^]^ were also highly expressed in hBM‐MK3 (Figure 1F; Figure S1H, Supporting Information). These results implied that the subpopulation of hBM‐MK3 might consist mainly of cells with strong potency to produce platelets. In contrast to other subpopulations, hBM‐MK4 exhibited enriched expression of mitochondrial genes associated with cellular respiration and ATP metabolism, including *MT‐ND4* (mitochondrially encoded NADH: ubiquinone oxidoreductase core subunit 4) (Figure 1E,F). Genes associated with organelle organization and transmembrane transport, such as *ICK* (ciliogenesis associated kinase 1), *AXIN1* (Axin 1), and *TMEM41B* (transmembrane protein 41B), were also enriched in hBM‐MK4 (Figure 1E,F; Figure S1H, Supporting Information). These MKs may be at the terminal stage and are primed for the assembly and release of platelets or microparticles. Interestingly, the hBM‐MK5 subpopulation, which constituted ≈7.1% of total cells, exhibited specific enrichment of immune‐associated gene sets, such as those with GO terms related to leukocyte activation, inflammatory response, and lymphocyte activation (Figure 1E,F; Figure S1‐H, Supporting Information).

Together, by modifying the MK isolation method and utilizing single‐cell profiling, we successfully isolated intact MKs from human native BM, established the single‐cell transcriptomic landscape, and revealed cellular heterogeneity of adult MKs for the first time.

### 2.2 Identification of a Subpopulation of “Immune MKs”

**Figure 2:**
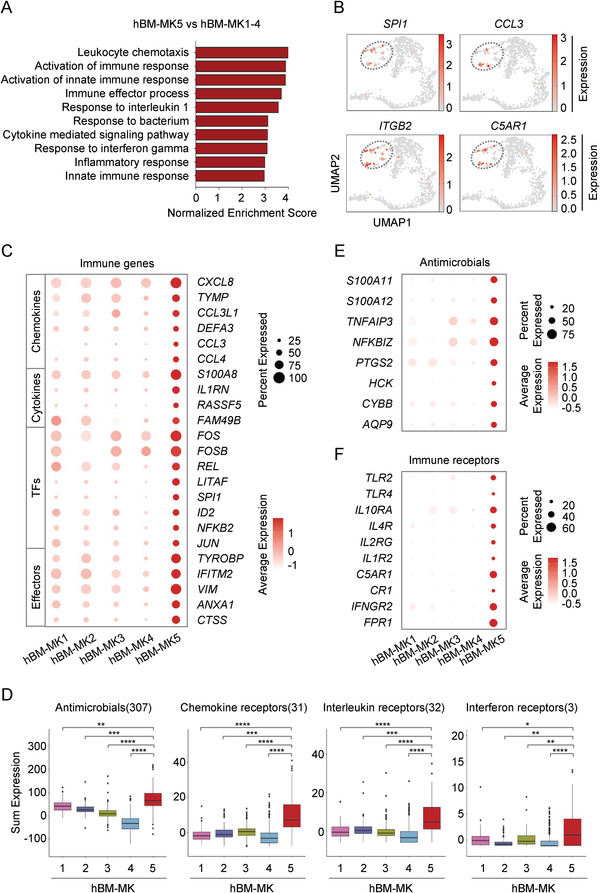
Identification of a subpopulation of “immune‐MKs.” A) Gene set enrichment analysis (GSEA) of immune gene sets enriched in hBM‐MK5 versus hBM‐MK1‐4. B) UMAP visualization of the expression of feature genes for hBM‐MK5. C) Dot plot showing the expression of various immune genes in five distinct MK clusters. D) Box plot displaying the expression level of genes related to antimicrobials and various immune receptors in each MK subset. Colors indicate different MK clusters described in Figure 1C. E) Dot plot showing the expression of representative genes of antimicrobials in human MK subsets. F) Dot plot showing the expression of multiple immune receptors in human MK subsets.

The enrichment of a large number of immune‐related genes in the hBM‐MK5 subpopulation (Figure 1E; Figure S1H, Supporting Information) led us to further determine how this subpopulation of MKs differs from other subpopulations, we pooled together the MK subpopulation 1 to 4 (termed hereafter as hBM‐MK1‐4) and then compared them with hBM‐MK5. Consistently, the top ten enriched gene sets in hBM‐MK5 were all associated with immune response in contrast to hBM‐MK1‐4 (**Figure** **2**A). Gene set enrichment analysis (GSEA) also revealed similar enrichments of genes associated with “leukocyte mediated immunity,” “cellular response to cytokine stimulus,” and “cellular response to interferon gamma” in hBM‐MK5 (Figure S2A, Supporting Information). Some critical genes associated with immune responses, such as *CCL3* (C‐C motif chemokine ligand 3), a potent activator of both innate and adaptive responses,^[^ ^21^ ^]^ and *SPI1* (Spi‐1 Proto‐Oncogene), a transcription factor important for B cell antigen receptor signaling,^[^ ^22^ ^]^ showed specific expression in the hBM‐MK5 subpopulation (Figure 2B). Thus, the main differentially expressed genes in hBM‐MK5, when compared with other MKs in adult BM, were all immune‐associated genes, and these genes could be further classified into distinct groups such as chemokines, cytokines, TFs, and immune effectors (Figure 2C).

From the prospective of cellular functions, a number of “antimicrobials associated genes,” such as multiple inflammatory mediators *S100A11* (S100 calcium binding protein A11), *S100A12* (S100 calcium binding protein A12), and *TNFAIP3* (TNF alpha induced protein 3),^[^ ^23^, ^24^ ^]^ were highly expressed in hBM‐MK5, but not in other subpopulations of hBM‐MKs (Figure 2D,E). The higher expression of chemokine and interleukin genes was also observed in hBM‐MK5 (Figure S2B, Supporting Information). In contrast, minimal expression of interferon genes was detected in all of the 5 MK subpopulations (Figure S2B, Supporting Information). Furthermore, many receptors associated with immune responses, such as chemokine receptors, interleukin receptors, and interferon receptors were also highly expressed in hBM‐MK5 (Figure 2D,F). For example, receptors critical for innate immune response, such as *TLR2* (Toll‐like receptor 2) and *TLR4* (Toll‐like receptor 4),^[^ ^25^ ^]^ were present in hBM‐MK5 but not other subpopulations of MKs (Figure 2F). Other immune‐associated receptors including *IFNGR2* (interferon gamma receptor 2), *IL10RA* (interleukin 10 receptor subunit alpha), *IL4R* (interleukin 4 receptor), and *IL1R2* (interleukin 1 receptor type 2), which are involved in immune responses and signal transduction,^[^ ^26^, ^27^, ^28^, ^29^ ^]^ were also enriched specifically in hBM‐MK5 (Figure 2F).

In summary, our results revealed a subpopulation of MKs from human adult BM highly enriched in genes associated with immune responses. The enrichment led us to speculate that they might transmit signals using various receptors on their surface and subsequently exert a variety of immune functions, such as those related to innate immune responses. Thus, we termed this subpopulation of MKs as “immune MKs” hereafter.

### 2.3 Identification of “Immune MKs” in Human Megakaryopoiesis In Vitro

After identifying the subpopulation of immune MKs from hBM, we asked how they might be generated. The limited number of derived MKs and the difficulty in assessing human megakaryopoiesis in hBM make it infeasible to address this process in vivo. Instead, cultured stem cells in vitro offer a powerful alternative model system. Recently, we reported that the human MKs can be generated sequentially from BM HSPCs, which are most relevant to MKs directly isolated from native BM.^[^ ^30^ ^]^ Specifically, 8 and 12 days were needed, respectively, for polyploid MKs and proplatelets to form from this in vitro model of megakaryopoiesis of human BM (hiBM) (Figure S3A, Supporting Information).

**Figure 3:**
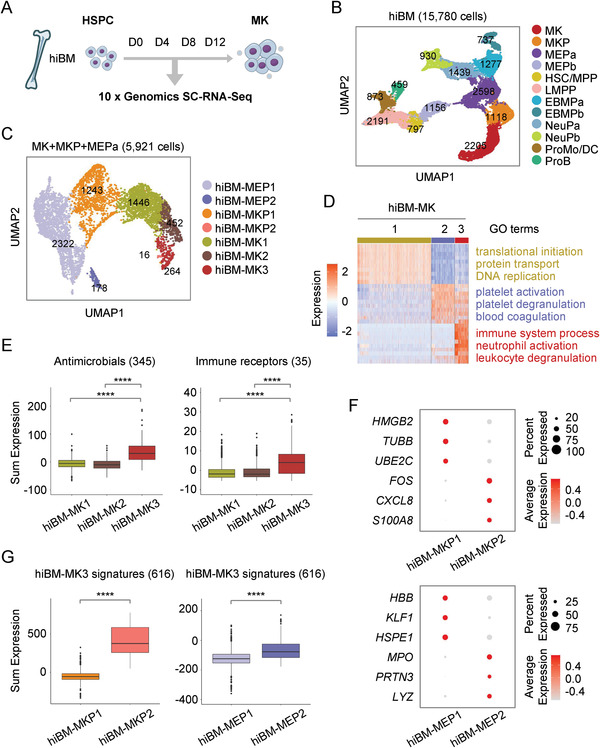
Identification of “immune MKs” in human megakaryopoiesis in vitro. A) Schematic diagram of single‐cell RNA‐seq cells collected from in vitro model of megakaryopoiesis from human BM (hiBM). B) Cell clusters of 15,780 single cells derived at all time points from hiBM model visualized by UMAP. Colors represent distinct cell types annotated by the expression of feature genes. Each dot represents one cell. C) The MK, MKP, and MEPa clusters identified in (B) were taken out for reclustering and visualized by UMAP. Subclusters were marked with different colors. D) Heatmap showing top ten differentially expressed genes in each subpopulation of mature MKs. Highlighted GO terms were selected by adjusted *P* value (<0.05). Colors indicate different MK clusters described in (C). E) Box plot displaying the expression level of genes related to antimicrobials and immune receptors in each MK subset. Colors indicate different MK clusters described in (C). F) Dot plot showing the expression of feature genes in each MKP and MEP subpopulation. G) Box plot showing the level of signature genes of immune MKs (hiBM‐MK3) in each MEP and MKP subsets. Colors indicate different MKP and MEP clusters described in (C).

We next performed single‐cell transcriptomic profiling to explore the path of human megakaryopoiesis. We collected various sets of cells derived at different time points and selected high‐quality intact single cells for RNA‐Seq using the 10x Genomics Chromium platform (**Figure** **3**A). After quality control, 15,780 cells in total, derived at all time points from the hiBM model, were selected for analysis (Figure 3B). More than 2,500 genes were analyzed in each cell. Cell populations were featured and annotated by using the expression of known marker genes.^[^ ^31^, ^32^ ^]^ Twelve populations in total, including the MK, MKP, MEP, HSC/MPP, lymphoid‐primed multipotent progenitor (LMPP), and other immune progenitors, were identified (Figure 3B). Among various clusters, the featured MK and MKP clusters increased during megakaryocytic differentiation (Figure S3B, Supporting Information). Specific expressions of *GP1BA* (CD42b), *PLEK* (Pleckstrin), and *GP9* (glycoprotein 9) in MK cluster were observed (Figure S3C, Supporting Information). Thus, we derived a population of bona fide MKs from human BM HSPCs and successfully revealed the single‐cell transcriptomic profiles.

We further asked whether the immune MKs could be generated during human megakaryopoiesis in vitro. To do this, we chose the in vitro generated MK, MKP, and MEPa clusters for further analysis. By using unsupervised clustering, we identified two subpopulations of MEPs, two subpopulations of MKPs and three distinct subsets of MKs, respectively (Figure 3C; Figure S3D, Supporting Information). Consistent with the maturation process of MKs, the expression of maturation‐related genes (Table S2, Supporting Information) was gradually elevated from MEPs, MKPs to mature MKs, which expectedly showed the highest maturation score (Figure S3E, Supporting Information). All of MKs generated from BM‐derived HSPCs were termed hereafter as hiBM‐MK1 to hiBM‐MK3 (Figure S3F, Supporting Information). Strikingly, a large number of genes associated with immune process, neutrophil activation, and leukocyte degranulation were found enriched in hiBM‐MK3 subpopulation (Figure 3D). In keeping with the observations from hBM‐MK5, many genes associated with antimicrobials or immune receptors were also highly enriched in MK3 (Figure 3E). Furthermore, the hiBM‐MK3 from in vitro cultures showed the best matching with hBM‐MK5 in vivo, as predicted by using the LabelTransfer function in Seurat (Figure S3G, Supporting Information). The immune signature genes of hBM‐MK5 were expressed much more highly in hiBM‐MK3 than in the other two subpopulations (Figure S3H, Supporting Information), further confirming the similarity of in vivo and in vitro derived MKs.

After confirming the existence of immune MKs in vitro, we further explored the origins of these immune MKs. Interestingly, a subset of MKP, termed as hiBM‐MKP2, exhibited strong immune features, as evidenced by the specific expression of genes related to immune response, such as inflammatory mediators *CXCL8* (C‐X‐C motif chemokine ligand 8)^[^ ^33^ ^]^ and *S100A8* (S100 calcium binding protein A8)^[^ ^34^ ^]^ (Figure 3F). The immune‐biased MEP subset, termed as hiBM‐MEP2, was also identified by the higher expression of antimicrobial genes *MPO* (myeloperoxidase)^[^ ^35^ ^]^ and LYZ (lysozyme)^[^ ^36^ ^]^ (Figure 3F). Furthermore, the immune signature genes of hiBM‐MK3 were expressed much more highly in hiBM‐MKP2 and hiBM‐MEP2 subpopulations than in other subsets (Figure 3G), demonstrating that the immune signatures of MKs could be traced back to an earlier stage.

### 2.4 CD148 and CD48 Mark the “Immune MKs”

**Figure 4:**
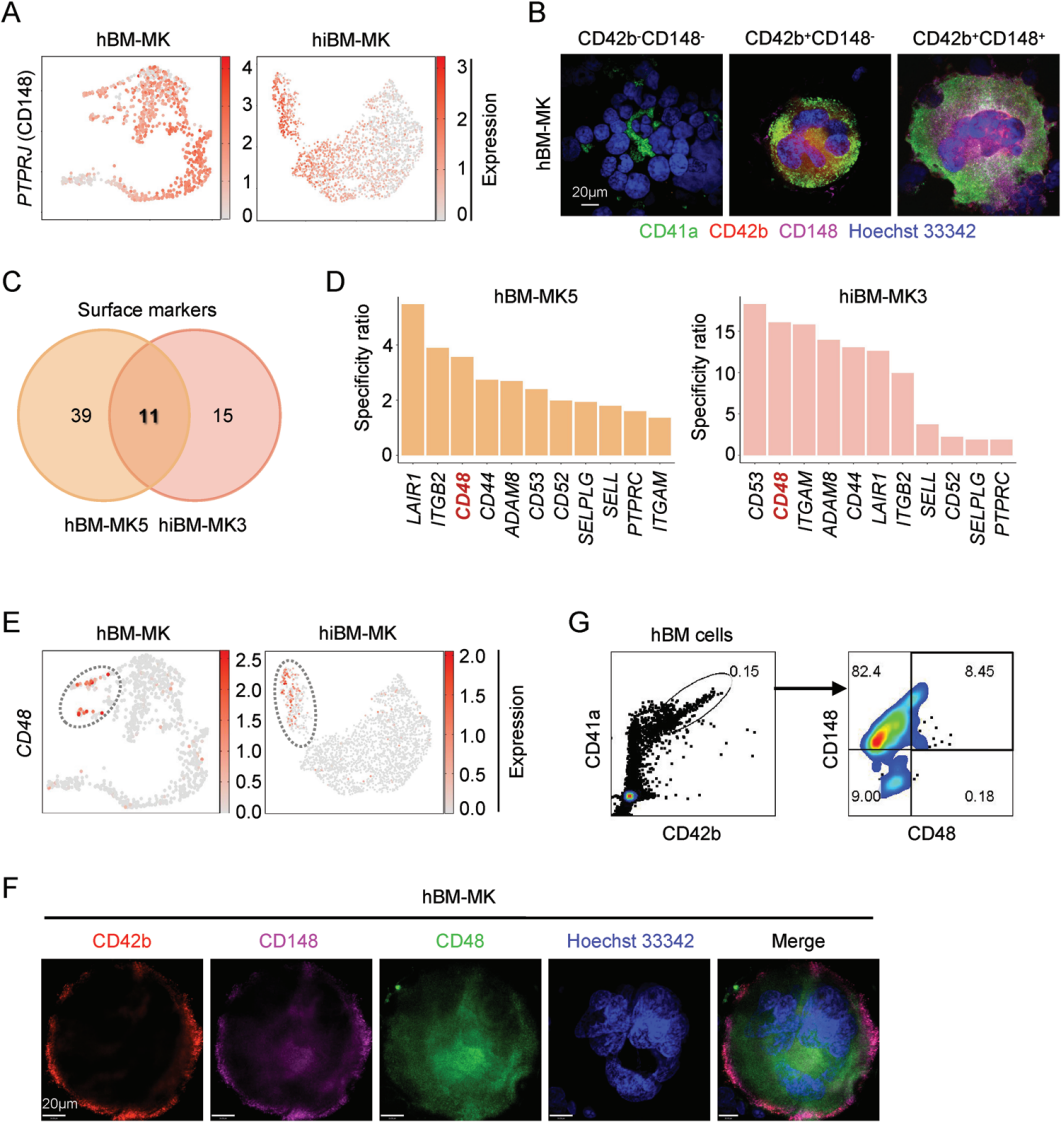
CD148 and CD48 mark the “immune MKs.” A) UMAP plot showing *PTPRJ* (CD148) expression in mature MKs both in vivo and in vitro. B) Typical morphologies of three types of MK populations detected by immunofluorescent staining of CD41a, CD42b, CD148, and Hoechst33342 on the BM smears of healthy donors (scale bar, 20 µm). C) Screening of potential surface markers that were common to immune MKs from both native BM and in vitro cultures. D) Candidate surface markers of immune MKs ranked by the specificity ratio (positive expression rate of candidate genes in MK populations vs progenitors) both in vivo and in vitro. E) UMAP plot showing *CD48* expression in immune MKs both in vivo and in vitro. F) The morphology of CD148^+^CD48^+^ MKs in the BM smears of healthy donors (scale bar, 20 µm). G) The percentage of CD148^+^CD48^+^ MKs gated in CD41a^+^CD42b^+^ cells in human BM measured with flow cytometry.

To further define the function of the immune MKs, it would be necessary to develop a method to isolate them from the MK population. This would require the identification of highly specific surface markers. As the first step toward achieving this goal, we sought to discover markers specific for mature MKs, because the previously established surface markers, such as CD41a, CD42b, and CD61, are not adequate due to the lack of specificity for a particular stage(s) of megakaryopoiesis and the presence over a broad window of MK development (e.g., from MKPs to MKs, or from diploid MKs to polyploid MKs).^[^ ^37^ ^]^ To identify novel surface markers for mature MKs, we first screened computationally the surface markers highly expressed in MK populations rather than in MEPs or MKPs (Figure S4A, Supporting Information). Interestingly, a gene termed as *PTPRJ* (protein tyrosine phosphatase receptor type J) (or CD148), which is reportedly essential for MK maturation and platelet generation,^[^ ^38^ ^]^ exhibited the most pronounced difference in mature MKs as opposed to the MEP and MKP populations (**Figure** **4**A; Figure S4B, Supporting Information). We then assessed the expression of CD148 in vivo using immunofluorescence studies. Indeed, CD148 was highly expressed in more mature MKs both from human and mouse BM (Figure 4B; Figure S4C, Supporting Information). Thus, in subsequent studies we used CD148 as a surface marker for mature MKs.

To further identify potential marker(s) that might be used to enrich immune MKs, we screened surface markers highly enriched in immune MKs generated from both hBM and the in vitro differentiation system (Figure S4D, Supporting Information). Interestingly, eleven candidate surface markers that were common in immune MKs from both native BM and in vitro cultures were identified (Figure 4C). Among them, CD48 was the top three specific candidates in recognizing immune MKs both in vivo and in vitro (Figure 4D,E). To further validate the specificity of CD48, we first measured its expression in immune MKs in vitro. We found that the CD48 was highly expressed in CD148^+^ populations derived from BM CD34^+^ cells (Figure S4E, Supporting Information). We next showed that CD148^+^CD48^+^ immune MKs could be detected in vivo in both human and mouse BM using immunofluorescence studies (Figure 4F; Figure S4F, Supporting Information). Flow cytometry assay further confirmed these results (Figure 4G; Figure S4G, Supporting Information). Thus, the identification of CD148 and CD48 allows us to isolate immune MKs specifically and provides important tools for further functional dissection of this MK subpopulation.

### 2.5 CD148^+^CD48^+^ “Immune MKs” Can Respond to Immune Stimuli

**Figure 5:**
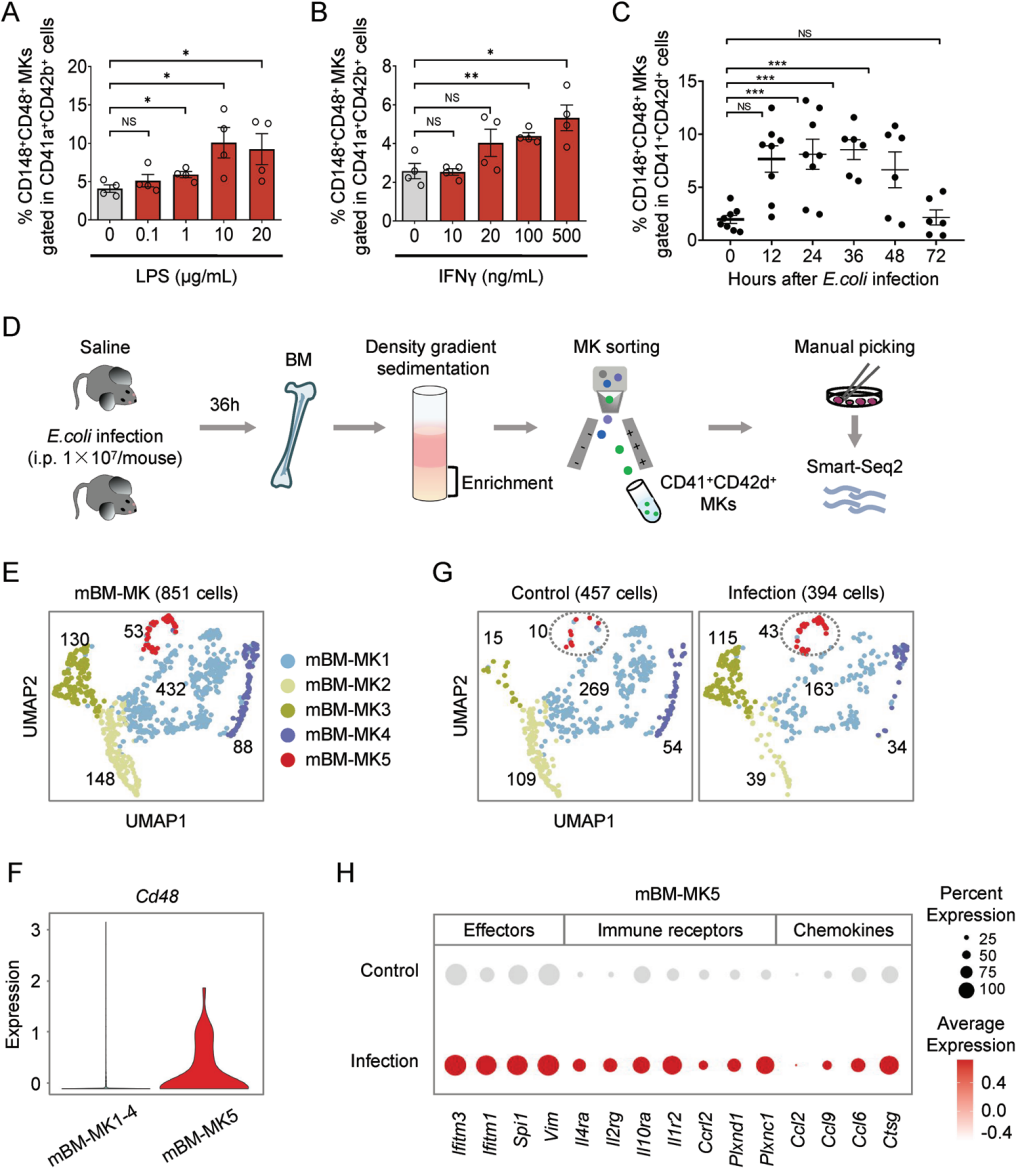
CD148^+^CD48^+^ “immune MKs” can respond to immune stimuli. A) The percentage of CD148^+^CD48^+^ subset gated in CD41a^+^CD42b^+^ MKs with stimulation of different concentrations of LPS. Data are pooled from four independent experiments (*n* = 4) and presented as mean ± SD. *P*‐values are calculated using a two‐tailed unpaired Student's *t* test, NS, not significant, ^*^ *P* < 0.05. B) The percentage of CD148^+^CD48^+^ subset gated in CD41a^+^CD42b^+^ with stimulation of different concentrations of IFN*γ*. Data are pooled from four independent experiments (*n* = 4) and presented as mean ± SD. *P*‐values are calculated using a two‐tailed unpaired Student's *t* test, NS, not significant, ^*^ *P* < 0.05 and ^**^ *P* < 0.01. C) The dynamic percentage of CD148^+^CD48^+^ immune subset gated in CD41^+^CD42d^+^ MKs in the BM of *E. coli*‐challenged mice within 72 h of infection. Data are pooled from 3 to 4 independent experiments with *n* = 4–8 mice per time point and presented as mean ± SD. *P*‐values are calculated using a two‐tailed unpaired Student's *t* test, NS, not significant, ^***^ *P* < 0.001. D) Schematic diagram of single‐cell RNA‐Seq of mouse MKs from the BM with or without *E. coli* infection. E) Cell clusters of 851 mouse MK single cells from two groups visualized by UMAP. Colors indicate different MK clusters. Each dot represents one cell. F) Violin plot showing the expression of *Cd48* in mBM‐MK5 versus mBM‐MK1‐4. G) The distribution of each MK subpopulation in control and infection groups visualized by UMAP. Colors indicate different MK clusters. Each dot represents one cell. H) Dot plot showing the expression of various immune genes in mBM‐MK5 in each group.

The identification of CD148^+^CD48^+^ immune MKs made it possible to further examine their functions. We first asked whether this subset of MKs could respond to various stress stimuli. Because of the high expression of TLR2 and TLR4 in immune MKs (Figure 2F) and the earlier observations that these immune receptors are implicated in signal transduction can be induced by lipopolysaccharide (LPS) in most Gram‐negative bacteria,^[^ ^25^ ^]^ we treated the cells undergoing megakaryocytic differentiation with different doses of LPS. Interestingly, although there was no significant change in the percentage of CD148^+^ MKs or the CD41a^+^CD42b^+^ cells, the fraction of CD148^+^CD48^+^ MKs was elevated 2 days after LPS stimulation in a concentration‐dependent manner (**Figure** **5**A; Figure S5A,B, Supporting Information). The enhanced production of CD148^+^CD48^+^ immune MKs was also observed in the presence of IFN‐*γ* (Figure 5B). These results indicated that the production of CD148^+^CD48^+^ immune MKs could be stimulated in response to immune stimuli in vitro.

We then determined whether CD148^+^CD48^+^ MKs could respond to immune stimuli in vivo. We used a mouse model to analyze the change of CD148^+^CD48^+^ MKs upon bacterial infection (Figure S5C, Supporting Information). We examined the dynamics of platelet production after the challenge of the mice with *Escherichia coli* (*E.coli*) and observed a sharp reduction in the number of platelets in the peripheral blood (PB) as early as 2 h after infection (Figure S5D, Supporting Information). The platelet number continued to decrease at 36 h but quickly recovered at 72 h after infection (Figure S5D, Supporting Information). Consistently, the percentage of CD148^+^ MKs decreased slightly within 48 h after bacterial infection, despite the weak increase of the proportion of CD41^+^CD42d^+^ cells in the mouse BM (Figure S5E,F, Supporting Information). Strikingly, the CD148^+^CD48^+^ MKs increased rapidly after 6 h of infection and peaked at 36 h when the platelet count was the lowest (Figure 5C). Finally, the proportion of CD148^+^CD48^+^ MKs returned to the steady‐state level (i.e., prior to infection) 72 h after bacterial infection, when the platelet count also returned to normal (Figure 5C). Thus, the CD148^+^CD48^+^ immune MKs can respond to various immune stimuli both in vitro and in vivo.

To understand the overall changes of individual MK subpopulations under inflammatory conditions, we collected CD41^+^CD42d^+^ cells in the mouse BM for Smart‐Seq2 sequencing 36 h after *E. coli* infection when the proportion of CD48^+^ MKs peaked (Figure 5D). After quality control was completed, we successfully obtained the integrated data of single‐cell transcriptomic profiling of 851 MKs from two groups, which both highly expressed *Itga2b*, *Gp5* (Cd42d), and *Ptprj*, well‐established markers for MKs (Figure 5E; Figure S5G,H, Supporting Information). Based on the clustering and cell annotation, 851 cells were divided into five subpopulations, named as mBM‐MK1 to mBM‐MK5, respectively (Figure 5E; Figure S5I, Supporting Information). Among them, genes associated with immune responses, such as *Mpo*, *Ccl3*, and S100A calcium‐binding proteins *S100a8* and *S100a9*, were specifically expressed in mBM‐MK5 (Figure S5I, Supporting Information), confirming the existence of mouse “immune MKs.” Consistent with our earlier results from the human and mouse models, the percentage of immune MK subpopulation (mBM‐MK5), which could also be identified through the specific expression of *Cd48*, increased substantially after *E. coli* infection (Figure 5F,G). Furthermore, the genes associated with inflammatory signals such as chemokines, chemokine receptors, interleukin receptors, and immune effectors showed higher expression in the mBM‐MK5 from infected mice (Figure 5H), indicating that the cells are indeed responsive to infections.

In addition to mBM‐MK5, we also observed changes in mBM‐MK2 and mBM‐MK3. After infections, a significant upregulation of mBM‐MK3 was observed, consistent with the change of mBM‐MK5. In contrast, mBM‐MK2 showed a rapid decrease (Figure 5G; Figure S5I, Supporting Information). These observations indicate that multiple MK subpopulations are involved in the immune response while different subpopulations of MKs might play distinct roles. The potential functions of different subpopulations of MKs await future studies.

### 2.6 The Functional Link between CD148^+^CD48^+^ MKs and Immune Surveillance

**Figure 6:**
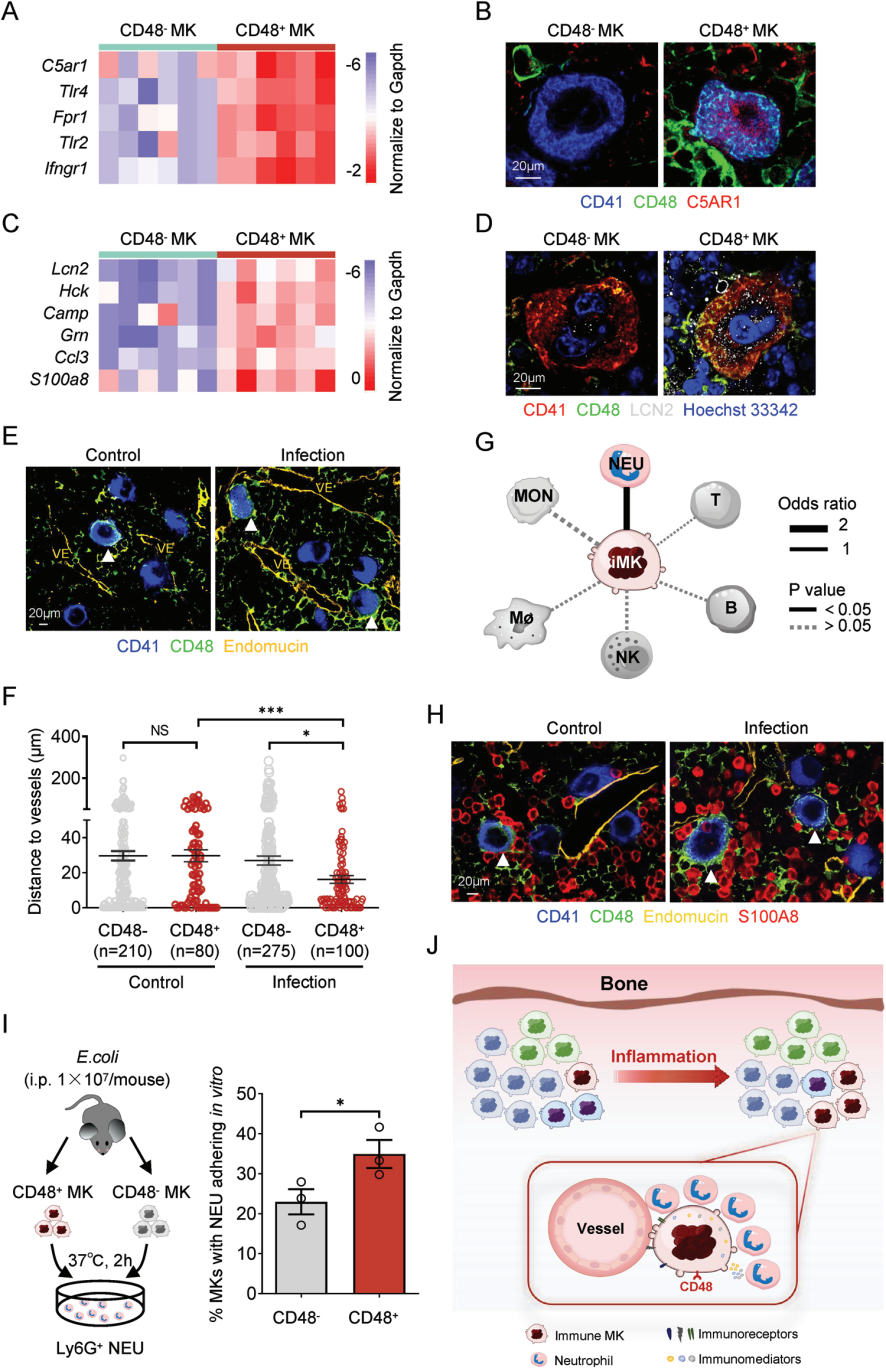
The functional link between CD148^+^CD48^+^ MKs and immune surveillance. A) The expression of representative immunoreceptors in CD48^+^ and CD48^−^ MKs measured with single‐cell qPCR. B) The expression of the immunoreceptor C5AR1 (CD88) in CD48^+^ and CD48^−^ MKs measured by in situ immunofluorescence staining (scale bar, 20 µm). C) The expression of representative immune mediators in CD48^+^ and CD48^−^ MKs measured with single‐cell qPCR. D) The expression of the immune mediator LCN2 in CD48^+^ and CD48^−^ MKs measured by in situ immunofluorescence staining (scale bar, 20 µm). E) The spatial relationship of CD48^+^ or CD48^−^ MKs and blood vessels (VE) in the BM of control or infected mice were detected by in situ immunofluorescence staining (scale bar, 20 µm). The CD48^+^ MKs were highlighted by white arrows and the blood vessels were stained by Endomucin in yellow. F) The shortest distance was calculated for CD48^+^ or CD48^−^ MKs to blood vessels in the BM of control (*n* = 290) or infected mice (*n* = 375). G) Networks of potential cell–cell interactions between immune MKs (iMK) and hematopoietic immune cells inferred from gene expression of known receptor–ligand pairs. NEU, neutrophil; MON, monocyte; Mø, macrophage; NK, natural killer cell; B, B cell; T, T cell. H) The interactions of CD48^+^ or CD48^−^ MKs with S100A8^hi^ neutrophils in the BM of control or infected mice were detected by in situ immunofluorescence staining (scale bar, 20 µm). The CD48^+^ MKs were highlighted by white arrows, and the blood vessels were stained by Endomucin in yellow. I) The percentage of sorted CD48^+^ or CD48^−^ MKs with Ly6G^+^ neutrophil adhesion after 2 h coculture at 37 °C. Data are pooled from three independent experiments (*n* = 3) and presented as mean ± SD. *P*‐values are calculated using a two‐tailed unpaired Student's *t* test, ^*^ *P* < 0.05. J) The schematic illustration of cellular heterogeneity of MKs and immune functions of CD48^+^ MK subpopulation. The MKs from native bone marrow consist of heterogeneous subpopulations. Among them, the immune MK subpopulation, which specifically expressed CD48, can respond to the inflammatory stimuli and increase substantially after infections. These CD48^+^ immune MKs, with high‐level expression of multiple receptors and mediators, might benefit from their short distance to the blood vessel after infections and function as potential immune sensors and modulators, which may contribute to neutrophil migration and mobilization.

The increase of the number of immune MKs after infections led us to dissect the role of the cells in acute inflammatory responses. Because both the hBM‐MK5 and mBM‐MK5 subpopulations expressed a plethora of immune receptors, including Toll‐like receptors, complement receptors, and inflammatory cytokine receptors (Figure 2F; Figure S6A, Supporting Information), we hypothesized that they might exert the function of immune surveillance to perceive the “signal‐of‐danger” and subsequently respond to inflammation rapidly. To test our hypothesis, we sorted the CD148^+^CD48^−^ and CD148^+^CD48^+^ MKs, respectively, from the immune‐challenged mice and performed single‐cell qPCR analysis, leading us to discover the high‐level expression of several pattern‐recognition receptors, including *C5ar1* (CD88), *Fpr1* (Formyl peptide receptor 1), and Toll‐like receptors *Tlr2* and *Tlr4* in CD48^+^ MKs (**Figure** **6**A). The expression of C5AR1 and TLR4 in CD48^+^ MKs was also confirmed by the use of in situ immunofluorescence staining and/or flow cytometry at the protein level (Figure 6B; Figure S6B, Supporting Information). In addition, we detected the expression of immune mediators, including *S100A8*, *CCL3*, and *LCN2* (Lipocalin 2), at both the transcriptomic and protein levels in the immune subpopulation of both human and mouse MKs (Figure 6C,D; Figure S6C,D, Supporting Information). Furthermore, we found that the average distance of CD48^+^ MKs to blood vessels (16.15 ± 0.22 µm) was substantially smaller than that of CD48^−^ MKs (26.92 ± 0.15 µm) while nearly a half of the CD48^+^ MKs were all localized in close proximity to blood vessels (<5 µm) in the mouse BM after immune challenges (Figure 6E,F; Figure S6E, Supporting Information). The high‐level expression of receptors and mediators, together with the close proximity to blood vessels, should allow CD48^+^ MKs to better exert the immune surveillance function during acute inflammation.

To explore how MKs communicate with the various immune cells in the BM, we examined potential cell–cell interactions based on known receptor–ligand pairs as previously described.^[^ ^39^ ^]^ Interestingly, the analysis of the cell–cell interaction network predicted a strong potential crosstalk between the immune MKs and neutrophils (Figure 6G; Table S3, Supporting Information). Because S100A8 (which forms a heterodimer complex with S100A9) comprised ≈45% of the cytoplasmic proteins in neutrophils,^[^ ^34^ ^]^ we thus used S100A8 as a cellular marker for neutrophils in the BM. To assess the potential cellular interactions in vivo, we counted the number of S100A8^hi^ neutrophils in close proximity to CD48^+^ and CD48^−^ MKs, respectively, and found that the CD48^+^ MKs exhibited a much higher potential to interact and/or recruit S100A8^hi^ neutrophils than CD48^−^ MKs under both homeostasis and infection conditions (Figure 6H; Figure S6F,G, Supporting Information). We further isolated CD48^−^ and CD48^+^ MKs from infected mice and cocultured them with Ly6G^+^ neutrophils in vitro, leading us to discover higher percentage of CD48^+^ MKs adhered directly to neutrophils than CD48^−^ MKs independently of other niche cells (Figure 6I; Figure S6H, Supporting Information). In summary, the immune MKs, which expressed high‐level expression of receptors and mediators, exhibited higher capacity to interact and/or recruit neutrophils and might function as immune‐surveillance cells during acute inflammation.

## 3 Discussion

In this study, we provided the single‐cell transcriptomic landscape of MKs from human BM and unveiled cellular heterogeneity of human adult MKs for the first time. Interestingly, a novel MK subpopulation that expresses a plethora of immune‐associated genes was identified. More importantly, these immune MKs, marked by CD148 and CD48, can respond to multiple immune stimuli both in vitro and in vivo. At the functional level, CD148^+^CD48^+^ MKs may benefit from their short distance to the blood vessel after infections and function as potential immune sensors and modulators, which may contribute to neutrophil migration and mobilization (Figure 6J).

The rarity and frangibility of human adult MKs have made the exploration of MKs and human megakaryopoiesis extremely challenging in vivo. To date, human MKs have been partially characterized at the transcriptomic level in bulk by using different hematopoietic differentiation models.^[^ ^40^, ^41^ ^]^ In addition, the current research has been focused on the number and size of MKs and platelets in the diagnosis of multiple platelet disorders, such as myelodysplastic syndromes (MDSs),^[^ ^42^ ^]^ myeloproliferative neoplasms (MPNs),^[^ ^43^, ^44^ ^]^ and immune thrombocytopenia (ITP),^[^ ^45^ ^]^ while little attention has been paid to the potential changes of MKs isolated from human native BM at the molecular level. In this study, we have broken through the technical bottleneck of MK isolation from human native BM and performed the single‐cell profiling of adult MKs for the first time. Recently, we decoded the human MKs from yolk sac and fetal liver in a single‐cell solution.^[^ ^14^ ^]^ These single‐cell transcriptomic analysis of both human embryonic and adult MKs have enabled us to identify numerous regulators of human megakaryopoiesis. It will thus provide invaluable resources for investigating the molecular mechanism of human MK development under various physiological and pathological conditions in future studies.

It has been well documented that mammalian adult MKs have diverse functions, including coagulation, homeostasis, angiogenesis, stem cell maintenance, and immune regulation.,^[^ ^8^, ^46^ ^]^ but it remains unclear whether the various functions are fulfilled by the entire MK populations or by distinct MK subpopulations. It was reported earlier that HIMeg‐1, a subclone of the human promegakaryoblastic cell line HIMeg, is capable of both monocytic and megakaryocytic differentiation when exposed to various agents.^[^ ^47^ ^]^ Furthermore, the administration of an activated form of tyrosyl‐tRNA synthetase variant in mouse BM induces the selective expansion of a unique MK supopulation expressing Sca‐1 and the macrophage marker F4/80, although the function of these MKs has not been assessed.^[^ ^48^ ^]^ These earlier studies provide fragmentary evidence to support the existence of specific MK subsets that may fulfill different functions. Recently, we showed by using single‐cell RNA sequencing that embryonic MKs from human yolk sac and fetal liver exhibit cellular heterogeneity.^[^ ^14^ ^]^ The results from the single‐cell profiling of human adult MKs in this study further confirm the existence of heterogeneous MK subpopulations in native BM. Together, our studies provide compelling evidence for the existence of cellular heterogeneity of human embryonic and adult MKs.

Evidence is emerging for the immune functions of MKs at both the phenotypical and functional levels.^[^ ^8^ ^]^ For example, it has been reported that MKs express many immune receptors including the members of TLR family and IgG Fc receptors.^[^ ^49^, ^50^, ^51^ ^]^ These receptors may enable MKs to sense inflammatory signals because the activation of TLR2 and TLR4 accelerates MK maturation and platelet production during the infection episodes.^[^ ^52^ ^]^ Furthermore, MKs produce pro‐inflammatory mediators or microparticles to mediate inflammatory and immunity reaction.^[^ ^53^, ^54^ ^]^ In addition, new studies showed that MKs from mouse lung exhibit an specific enrichment of immune molecules and skew toward roles in microbial surveillance and antigen presentation.^[^ ^12^, ^55^ ^]^ Interestingly, we recently demonstrated that the immune‐associated genes are highly expressed in a specific subpopulation of MKs from human yolk sac and fetal liver, instead of distributing equally in all embryonic MKs, which appear to be generated along a distinct route.^[^ ^14^ ^]^ In the current study, we also identified the immune subpopulation of human BM MKs with high expression of many immune receptors and proinflammatory mediators, which play the potential role of immune surveillance during acute inflammation. Furthermore, the specific enrichment of immune signatures in progenitor cells was also observed. All these observations lead us to speculate that the immune MKs indeed exist in various developmental stages of mammals and are likely generated along a distinct developmental route. A more comprehensive understanding of the precise functions of the immune MKs awaits future experimentation.

By using bioinformatic screening and functional validations, we have identified two cell surface markers, CD148 and CD48, which can be used as effective tools to isolate distinct functional subsets of human mature MKs. The function of CD148 in platelet generation and activation has been established.^[^ ^38^, ^56^ ^]^ In this study, we extended the previous findings and showed that CD148 expression occurs later than CD42 and can thus be used as a valuable marker for the enrichment of more “mature” MKs in human BM with higher polyploidy and potency for platelet generation. The expression of CD48 and its function have been well investigated in many immune cells, such as monocytes and T cells.^[^ ^57^ ^]^ In this study, we found that CD48 is present in adult MKs with immune characteristics. Experiments with these two surface markers, CD148 and CD48, enabled us to identify a CD148^+^CD48^+^ subpopulation of human adult MKs with strong immune characteristics that can respond to multiple immune challenges. In our earlier study, by decoding the cellular heterogeneity of human embryonic MKs, we identified a subpopulation of CD14^+^ MKs with high expression of immune‐related genes.^[^ ^14^ ^]^ The differences in the cellular markers between embryonic and adult immune MKs probably imply the functional distinctions between them. This interesting possibility should be further explored in the future.

In addition, future experimental efforts should be made to reveal the precise function of the distinct subpopulations of MKs, using functional assays such as cell transplantation and/or depletion. Furthermore, as the progenitor cells of platelets, whether the cellular heterogeneity of mature MKs might also lead to the heterogeneity of platelets is an interesting topic for exploration and discovery.

## 4 Experimental Section

### Experimental Models and Subjects

Human BM samples were obtained with informed consent after approval by the ethical committee of Institute of Hematology and Blood Diseases Hospital, Chinese Academy of Medical Sciences University. C57BL/6 mice were purchased from the State Key Laboratory of Experimental Hematology (SKLEH), Institute of Hematology and Blood Diseases Hospital, Chinese Academy of Medical Sciences University. All mouse experiments were approved by the Animal Care and Use Committee of the SKLEH and conformed to the relevant regulatory standards.

### MK Isolation from Human and Mouse Native BM

Human MKs were purified from the native BM by using a modified method of unit velocity albumin gradients and fluorescence‐activated cell sorting.^[^ ^15^ ^]^ Briefly, human BM cells were first mixed with ACK lysis buffer (1:3, Beyotime) to remove the red blood cells. Large cells were enriched by the density gradient sedimentation of bovine serum albumin for 40 min and stained with antihuman APC‐CD41a and PE‐CD42b antibodies in Dulbecco's phosphate‐buffered saline (DPBE with 2% fetal bovine serum and 2 mM EDTA) at 4 °C for 30 min in the dark. The CD41a^+^CD42b^+^ MKs were sorted using a FACS AriaIII flow cytometer (BD Biosciences) with a 130 µm nozzle. The CD41^+^CD42d^+^ MKs from the BM of *E. coli*‐challenged (36 h) and control mice were also isolated with the same method.

### Human Megakaryocytic Differentiation In Vitro

Megakaryocytic differentiation of CD34^+^ cells isolated from BM samples was performed as previously described.^[^ ^30^ ^]^ Briefly, the previously enriched CD34^+^ cells were first cultured in the serum‐free medium (StemSpan SFEM) containing 1% penicillin/streptomycin, thrombopoietin (TPO, 50 ng mL^−1^), stem cell factor (SCF, 20 ng mL^−1^), and interleukin‐3 (IL‐3, 20 ng mL^−1^) for 6 days and subsequently in StemSpan SFEM medium supplemented with TPO (50 ng mL^−1^) and interleukin‐11 (IL‐11, 20 ng mL^−1^, PeproTech) for another 6 days. Fresh medium was changed every 3 days.

### Single‐Cell RNA‐Seq

The single‐cell RNA‐seq library preparation and sequencing were performed based on the modified Smart‐Seq2 protocol.^[^ ^58^ ^]^ For human MKs isolated in vivo, the single CD41a^+^CD42b^+^ cells were transferred into lysis buffer by manual picking and sequenced on Illumina Hiseq X ten platform. For mouse MKs isolated in vivo, the single CD41^+^CD42d^+^ cells were treated with the same Smart‐Seq2 protocol and sequenced on NovaSeq 6000 platform (Novogene). For human MKs differentiated in vitro, total cells on day 0, day 4, day 8, and day 12 of MK differentiation from BM‐CD34^+^ HSPCs were collected and used to perform the single‐cell RNA‐Seq using 10x Genomics Chromium platform (Novogene). All the raw data of single‐cell RNA‐seq are deposited at National Omics Data Encyclopedia (NODE) with accession codes OEP000756, OEP001150, and OEP001128. The detailed information about the quality control and data processing is described in the Experimental Section in the Supporting Information.

### Statistical Analysis

Statistical analyses were performed with the GraphPad Prism 8.0 (GraphPad Software, Inc., CA, USA). For all cellular experiments in vitro, three or four independent replicates were performed and the number of biological replicates was indicated by the *n* value. For all in vivo analysis, three or four independent replicates were performed and a reasonable sample size was also chosen in each independent experiment. Mice were randomized prior to treatment and the number of mice at each time point was indicated by the *n* value. All graphs depicted mean ± SD. All statistical procedures were performed using a two‐tailed unpaired Student's *t* test, and the results were considered statistically significant at *P* value <0.05 and were denoted as NS, not significant; ^*^ *P* < 0.05; ^**^ *P* < 0.01; ^***^ *P* < 0.001.

## Conflict of Interest

The authors declare no conflict of interest.

## Author Contributions

C.L., D.W., and M.X. contributed equally to this work. J.Z., C.L., D.W., M.X., X.G., and T.C. coordinated and designed the project. C.L. and M.X. did the single‐cell RNA‐Seq. D.W., M.L., and X.G. did the single‐cell RNA‐seq analysis. C.L. and M.X. performed experiments and data acquisition. E.J., J.Z., Y.L., Z.S., M.W., and B.S. provided and collected the clinical samples. Q.W., H.W., P.S., L.S., Z.X., and W.Z. provided new thought and method. J.Z., C.L., X.G., and T.C. wrote, reviewed, and edited the manuscript.

## Supporting information

Supporting Information

## Data Availability

All the raw data of single‐cell RNA‐seq are deposited at National Omics Data Encyclopedia (NODE) with accession code OEP000756, OEP001150 and OEP001128.
