# Supplementary material for: Characterization of Cellular Heterogeneity and an Immune Subpopulation of Human Megakaryocytes
Source: Adv Sci (Weinh). 2021 May 26;8(15):2100921. doi: 10.1002/advs.202100921 (PMC8336508; doi:10.1002/advs.202100921)
Supplement: Supplementary file 1 — Supporting Information [file ADVS-8-2100921-s001.pdf]

© 2021 Wiley-VCH GmbH

ADVANCED SCIENCE Open Access

Supporting Information

for *Adv. Sci.,* DOI: 10.1002/advs.202100921

Characterization of Cellular Heterogeneity and an Immune Subpopulation of Human Megakaryocytes

Cuicui Liu, Dan Wu, Meijuan Xia, Minmin Li, Zhiqiang Sun, Biao Shen, Yiying Liu, Erlie Jiang, Hongtao Wang, Pei Su, Lihong Shi, Zhijian Xiao, Xiaofan Zhu, Wen Zhou, Qianfei Wang, Xin Gao, Tao Cheng*, Jiaxi Zhou**

Supporting Information

Characterization of Cellular Heterogeneity and an Immune Subpopulation of Human Megakaryocytes

Cuicui Liu[1,2,†], Dan Wu[1,2,†], Meijuan Xia[1,2,†], Minmin Li[1,2], Zhiqiang Sun[1,2], Biao Shen[1,2], Yiying Liu[1,2], Erlie Jiang[1,2], Hongtao Wang[1,2], Pei Su[1,2], Lihong Shi[1,2], Zhijian Xiao[1,2], Xiaofan Zhu[1,2], Wen Zhou[3], Qianfei Wang[4], Xin Gao[1,2,*], Tao Cheng[1,2,*], Jiaxi Zhou[1,2,*]

[1]State Key Laboratory of Experimental Hematology, National Clinical Research Center for Blood Diseases, Institute of Hematology and Blood Diseases Hospital, Chinese Academy of Medical Sciences and Peking Union Medical College, Tianjin 300020, China;

[2]Center for Stem Cell Medicine, Chinese Academy of Medical Sciences and Department of Stem Cells and Regenerative Medicine, Peking Union Medical College, Tianjin, 300020, China;

[3]Key Laboratory of Carcinogenesis and Cancer Invasion, Ministry of Education; Key Laboratory of Carcinogenesis, National Health and Family Planning Commission; Cancer Research Institute, School of Basic Medical Science, Central South University, Changsha, 410078, China;

[4]Key Laboratory of Genomic and Precision Medicine, Collaborative Innovation Center of Genetics and Development, Beijing Institute of Genomics, Chinese Academy of Sciences, Beijing 100101, China;

[†]equal contribution; [*]Corresponding authors: gaoxin1@ihcams.ac.cn; chengtao@ihcams.ac.cn; zhoujx@ihcams.ac.cn

Supporting information includes six supplemental figures, supplemental figure legends, five supplemental tables and supplemental experimental section.

Figure S1

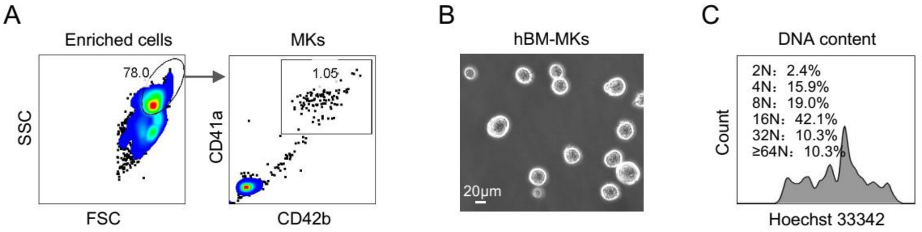

D

Sample	Age	Tissue	Sorting markers	Cell numbers
HD1	31	BM	CD41a⁺CD42b⁺	220
HD2	42	BM	CD41a⁺CD42b⁺	188
HD3	29	BM	CD41a⁺CD42b⁺	196
HD4	49	BM	CD41a⁺CD42b⁺	102
HD5	34	BM	CD41a⁺CD42b⁺	212
HD6	48	BM	CD41a⁺CD42b⁺	102
HD7	15	BM	CD41a⁺CD42b⁺	48
HD8	18	BM	CD41a⁺CD42b⁺	38

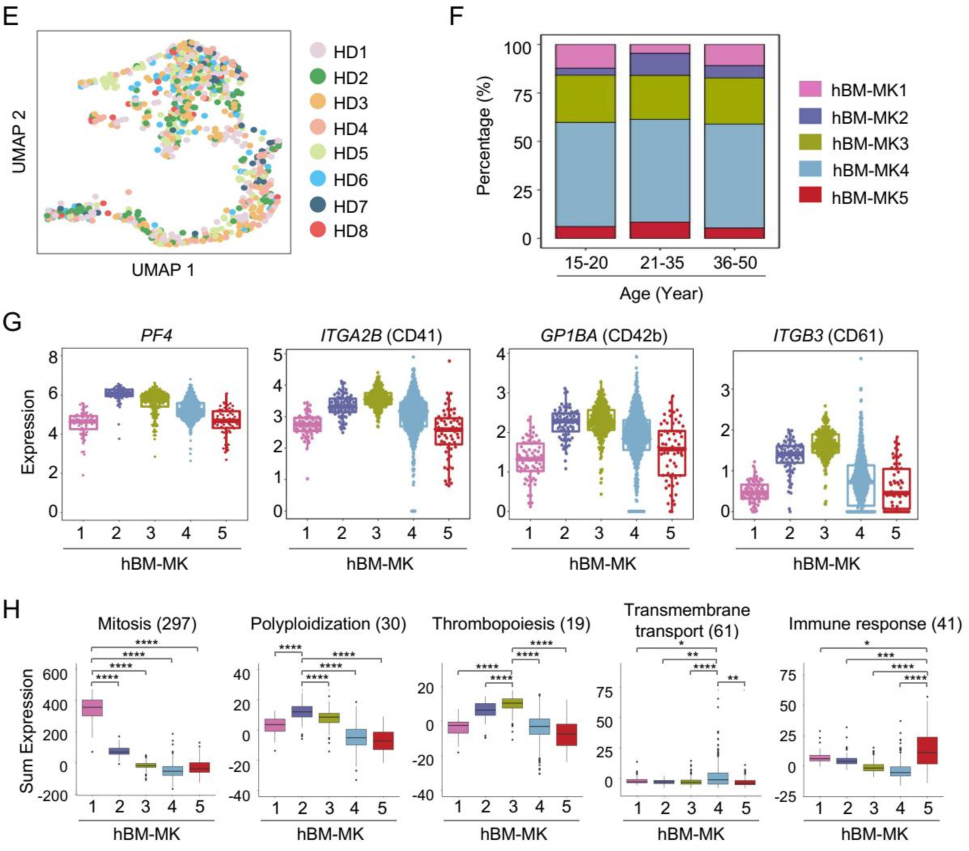

Figure S1 Single-cell profiling of human MKs from native BM

(A) Flow cytometry analysis of CD41a⁺CD42b⁺ MKs in human BM after BSA gradient enrichment.

(B) Representative morphologies of human MKs isolated from native BM (scale bar, 20 μm).

(C) The DNA content of human MKs from the BM was analyzed with flow cytometry.

(D) The summary of the information about human samples used for scRNA-seq profiling in this study.

(E) UMAP plot showing the cell distribution of human MKs from each sample.

(F) The proportional distribution of MK subpopulations in different age cohorts.

(G) Beeswarm plots of the expression of known marker genes for MK clusters. Colors indicate different MK subpopulations described in Figure 1C.

(H) Box plot showing the expression level of different gene sets in MK clusters. Colors indicate different MK subpopulations described in Figure 1C.

Figure S2

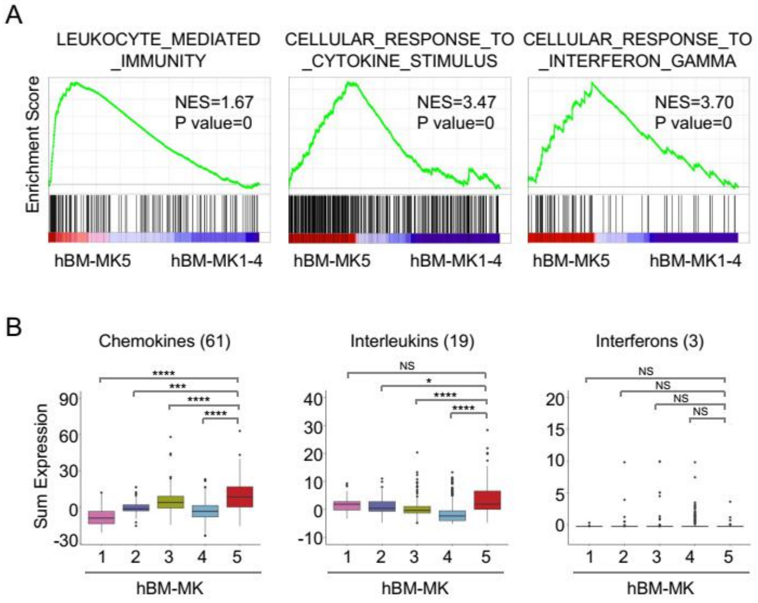

Figure S2 Identification of a subpopulation of "immune-MKs"

(A) GSEA plot showing that immune gene sets "leukocyte mediated immunity", "cellular response to cytokine stimulus" and "cellular response to interferon gamma" were enriched in hBMMK5 versus hBMMK1-4.

(B) Box plot displaying the expression level of genes related to various immune mediators in each MK subset. Colors indicate different MK clusters described in Figure 1C.

Figure S3

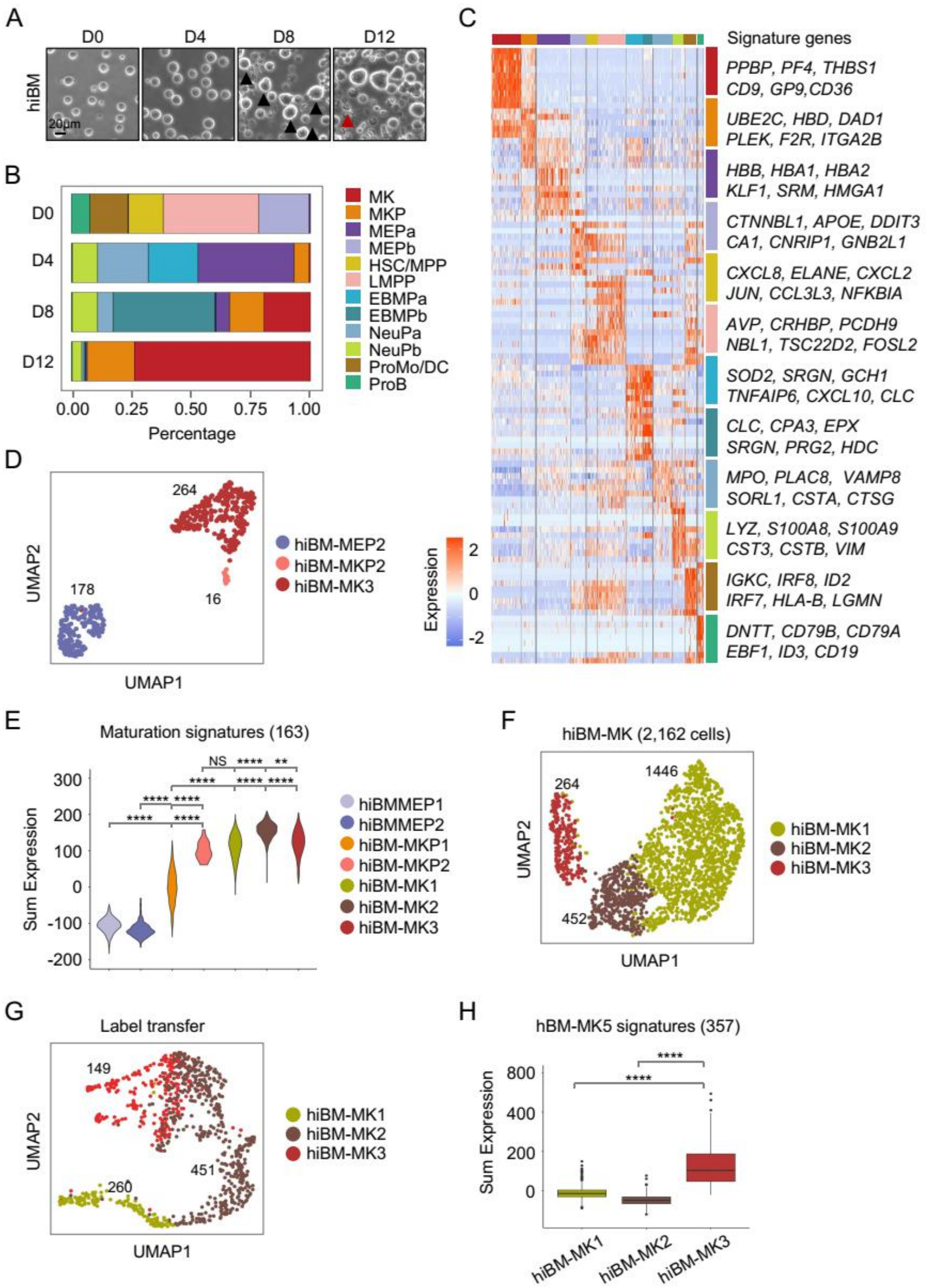

Figure S3 Identification of "immune MKs" in human megakaryopoiesis in vitro

(A) Morphological changes of megakaryocytic differentiation from human BM-derived HSPCs (scale bar, 20 μm). Black arrows denote larger MKs, and red arrows denote proplatelet-forming MKs.

(B) Cell composition in hiBM cultures at different timepoints. Colors represent distinct cell types described in Figure 3C.

(C) Heatmap showing top 10 highly differentially expressed genes in each cell cluster. The representative genes were displayed in the right panel.

(D) The immune-related subpopulations identified in Figure 3C (hiBM-MEP2, hiBM-MKP2 and hiBM-MK3) were visualized by UMAP.

(E) Box plot displaying maturation scores for each cluster based on the expression of MK maturation related genes (Table S2).

(F) Cell clusters of 2,162 single MKs derived from hiBM model visualized by UMAP. Colors represent distinct subpopulations of MKs. Each dot represents one cell.

(G) UMAP showing the distribution of each MK subpopulation in vitro when matched with in vivo MKs predicted by Label Transfer.

(H) Box plot showing the expression level of signature genes of immune MKs in vivo (hBMMK5) in each MK subsets in vitro.

Figure S4

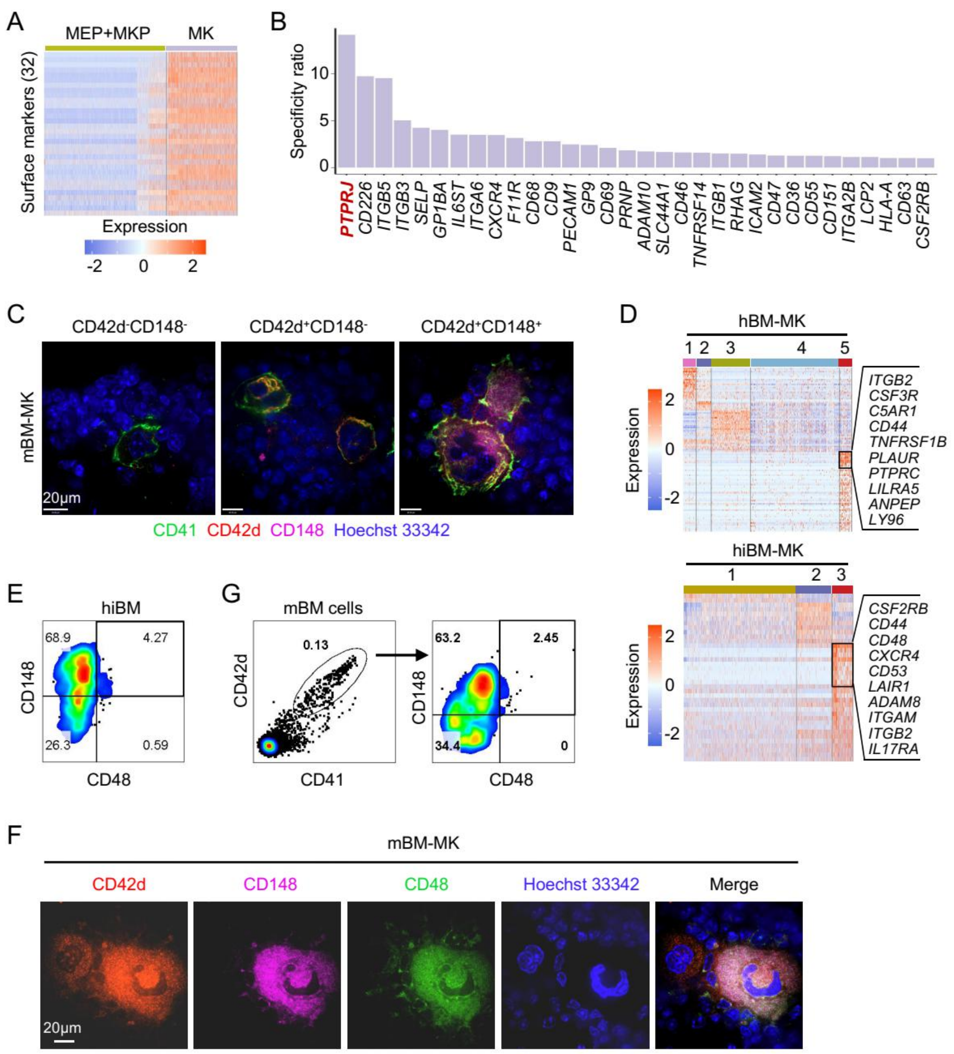

Figure S4 CD148 and CD48 mark the "immune MKs"

(A) Heatmap showing significantly differentially expressed surface markers in mature MKs versus progenitors.

(B) Candidate surface markers of mature MKs ranked by the specificity ratio (positive expression rate of candidate genes in MK populations versus progenitors).

(C) Typical morphologies of three types of MK populations detected with immunofluorescent staining of CD41, CD42d, CD148 and Hoechst33342 in the frozen sections of mouse BM (scale bar, 20 μm)

(D) Heatmap showing the differentially expressed surface markers in different MK subsets both in vivo and in vitro. The top 10 surface markers for immune MKs in both groups were listed in the right panel.

(E) The percentage of CD148^{+}CD48^{+} MKs gated in CD41a^{+}CD42b^{+} cells detected with flow cytometry in hiBM model.

(F) The morphologies of $CD148^+CD48^+$ MKs in the frozen sections of mouse BM (scale bar, 20 μm).

(G) The percentage of $CD148^+CD48^+$ MKs gated in $CD41^+CD42d^+$ cells in mouse BM measured with flow cytometry.

Figure S5

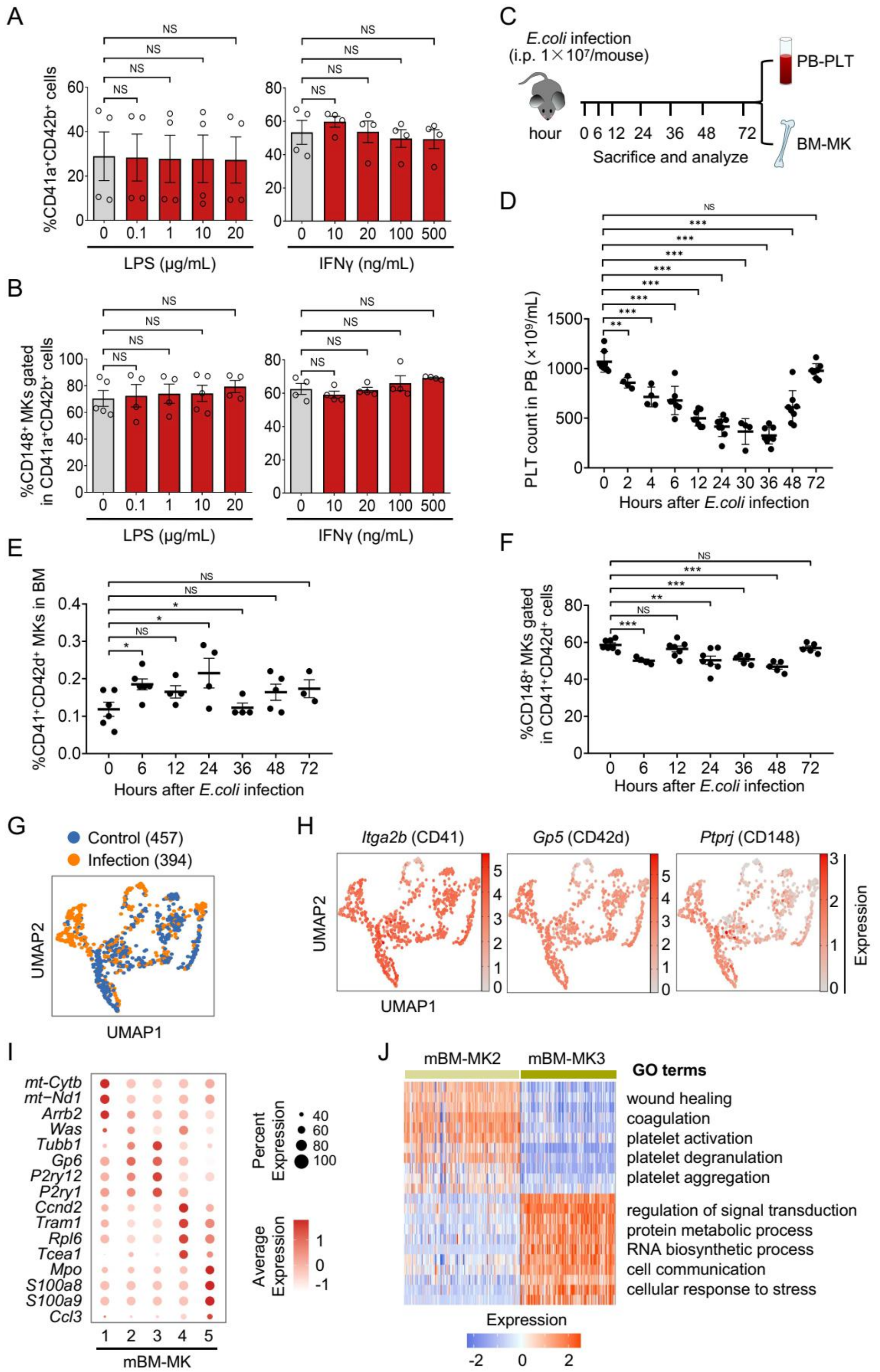

Figure S5 CD148⁺CD48⁺ "immune MKs" can respond to immune stimuli

(A) The percentage of $CD41a^+CD42b^+$ cells in CB model with the stimulation of different concentrations of LPS or IFNγ. Data are pooled from 4 independent experiments (n=4) and presented as mean ± SD. P-values are calculated using a two-tailed unpaired Student's t test, NS, not significant.

(B) The percentage of $CD148^+$ mature MKs gated in $CD41a^+CD42b^+$ cells with the stimulation of different concentrations of LPS or IFNγ. Data are pooled from 4 independent experiments (n=4) and presented as mean ± SD. P-values are calculated using a two-tailed unpaired Student’s t test, NS, not significant.

(C) Experimental scheme of the sample collection procedures after E.coli challenge.

(D) Platelet counts in the peripheral blood (PB) of mice measured at different time points after E. coli challenge. Data were pooled from 3-4 independent experiments with n=4-8 mice per time point and presented as mean ± SD. P-values are calculated using a two-tailed unpaired Student’s t test, NS, not significant, **P<0.01, ***P<0.001.

(E) The dynamics percentage of $CD41^+CD42d^+$ cells in the BM of E.coli-challenged mice within 72 hours of infection. Data were pooled from 3-4 independent experiments with n=3-6 mice per time point and presented as mean ± SD. P-values are calculated using a two-tailed unpaired Student’s t test, NS, not significant, *P<0.05.

(F) The dynamics percentage of $CD148^+$ MKs gated in $CD41^+CD42d^+$ cells in the BM of E.coli-challenged mice within 72 hours of infection. Data were pooled from 3-4 independent experiments with n=4-7 mice per time point and presented as mean ± SD. P-values are calculated using a two-tailed unpaired Student’s t test, NS, not significant, **P<0.01, ***P<0.001.

(G) UMAP plot showing the distribution of 851 mouse MKs in each group (457 cells from control mice and 394 cells from infection mice).

(H) UMAP visualization of the expression of known marker genes for mouse MK clusters.

(I) Dot plot showing the expression of feature genes in 5 distinct MK subpopulations.

(J) Heatmap showing the transcriptional profile of mBMMK2 versus mBMMK3. Highlighted GO terms were selected by adjusted P value (<0.05).

Figure S6

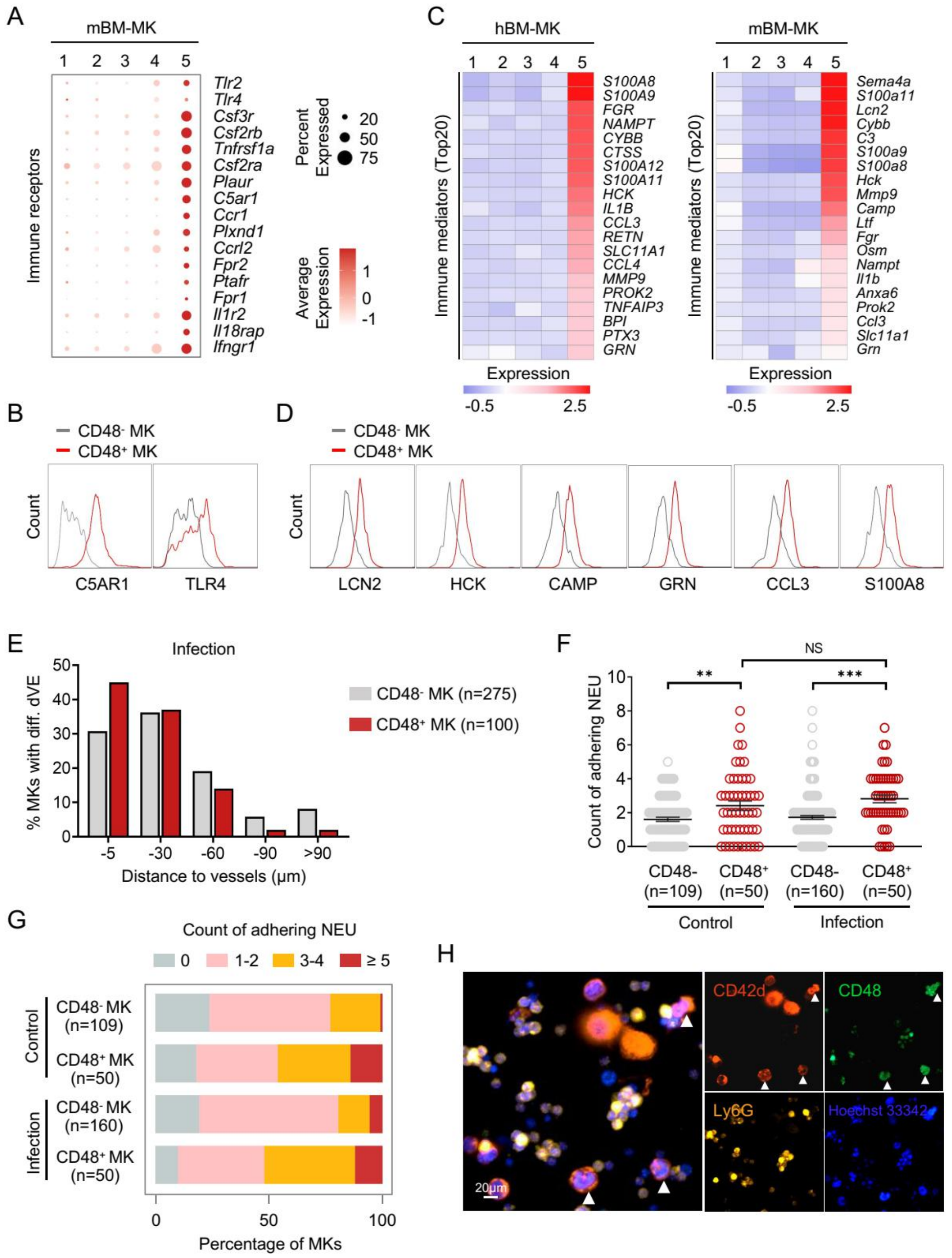

Figure S6 The functional link between CD148^{+}CD48^{+} MKs and immune surveillance

(A) Dot plot showing the expression of multiple immunoreceptors in mouse MK subsets.

(B) The expression of the immunoreceptors C5AR1 and TLR4 in CD48^{+} and CD48^{-} MKs as measured by flow cytometry.

(C) Heatmap showing the immune mediators enriched in the human and mouse immune subpopulation versus other MK subsets.

(D) The level of the immune mediators LCN2, HCK, CAMP, GRN, CCL3 and S100A8 in CD48^{+} and CD48^{-} MKs as measured by flow cytometry.

(E) The proportional distribution of $CD48^+$ (n=100) and $CD48^-$ MKs (n=275) with different distances to blood vessels (dVE) in the BM of infected mice.

(F) The number of $S100A8^{hi}$ neutrophils with direct adhesion to $CD48^+$ or $CD48^-$ MKs in the BM of control (n=259) and infected mice (n=210) were calculated on the basis of in situ immunofluorescence staining.

(G) The proportional distribution of MKs in close proximity to different number of $S100A8^{hi}$ neutrophils were calculated on the basis of in situ immunofluorescence staining.

(H) Cellular adhesion between sorted MKs and neutrophils in vitro detected with immunofluorescent staining of CD42d, CD48, Ly6G and Hoechst33342 (scale bar, 20 μm). The $CD48^+$ MKs were highlighted by white arrows.

Table S1 Genes involved in polyploidization and thrombopoiesis.[66-68]

Polyploidization		Thrombopoiesis
CCND1		
GABRA6	*CAV2*	
GPR87	*DDX53*	*ACTN1*
RHOQ	*UBE2E1*	*ARMC6*
FLJ31951	*TRAM1*	*CYCS*
CYP11B2	*SPAG1*	*FLNA*
ATP11A	*APRIN*	*GP9*
DMN	*RFP2*	*GP1BA*
NKX2-3	*NEK1*	*GP1BB*
EGFL6	*MARCH2*	*ITGA2B*
NCKAP1	*PLCB4*	*ITGB3*
CYP4V2	*CD96*	*MYH9*
LRRC59	*CALD1*	*WIPF1*
RAB10	*HLA-H*	*NBEAL2*
C14orf48	*ST3GAL6*	*TUBB1*
LOC283658	*ACTB*	*VWF*
KLK2	*ZFPM1*	*WAS*
FZD2	*MPL*	*PRKACG*
ZNF431	*FLI1*	*GP6*
GPR155	*VWF*	*P2RY12*
PCDH21	*ITGA2B*	*P2RY1*
MFAP3L	*ITGB3*	*MXD1*

Table S2 Genes related to MK maturation.[40]

IFI27	*THBS1*	*ACRBP*	*VCAN*	*TSC22D1*	*DLK1*
PTCRA	*LHFPL2*	*MTSS1L*	*TMEM185A*	*ARRDC4*	*CNST*
TFPI2	*SPDYC*	*HLA-DRA*	*MIR3190*	*HSPA1B*	*ITGB3*
ABCC3	*DGKD*	*TSPAN33*	*MIR25*	*PRKCA*	*CMTM5*
COL24A1	*ELOVL7*	*RASA3*	*INF2*	*NFKBIA*	*CASS4*
EHD3	*PDZK1IP1*	*ZNF185*	*FLNA*	*HLA-A*	*LY6G6E*
PTGIR	*ADCY6*	*STOM*	*PRKAR2B*	*HLA-H*	*C19orf33*
SLA2	*MYLK*	*VCL*	*LCN2*	*CTTN*	*LGALSL*
SELP	*CXCL3*	*PRUNE*	*ECE1*	*TSPYL2*	*MYZAP*
MEIS1	*C6orf25*	*KIF2A*	*NCK2*	*ABCC4*	*INAFM2*
CD226	*MAP3K5*	*GNB5*	*ALOX12*	*CCL5*	*ENDOD1*
GNG11	*LY6G6F*	*EMILIN1*	*HERC2P3*	*ZNF24*	*PSMB8-AS1*
SDPR	*LRRC8B*	*ITGA2B*	*SLC22A17*	*NCKAP1*	*TMEM63A*
RHOBTB1	*TSPAN18*	*TUBB1*	*FYB*	*TMEM140*	*HLA-B*
EGF	*SIAE*	*EPB41L3*	*ITGB5*	*BST2*	*ARHGAP18*
FRMD4B	*HPSE*	*GP1BB*	*KAT6A*	*LIMS1*	*PECAM1*
GJA4	*ABLIM1*	*PLEKHO1*	*CDKN1A*	*C1orf116*	*PDGFC*
ABLIM3	*PCSK6*	*SH3BP5-AS1*	*ATP2C1*	*SPP1*	*CD9*
VSIG2	*IFITM3*	*MIR142*	*SLC37A1*	*ACCS*	*C3orf58*
GP5	*HIST1H4H*	*UBASH3B*	*DUSP5*	*PEAR1*	*SERPINE2*
SPARC	*CTDSPL*	*ANO6*	*MAPRE2*	*GRAP2*	*NT5C3A*
CCR4	*F2RL3*	*BMP6*	*CMIP*	*TUBA4A*	*PSRC1*
NRGN	*GPNMB*	*TPM1*	*SYTL4*	*SLCO2B1*	*PBX1*

PPBP	*GP6*	*LYVE1*	*ICAM2*	*RUFY1*	*LTBP1*
RAB27B	*SERPINE1*	*MYL9*	*EGLN3*	*GABRE*	*GP1BA*
MMD	*TIMP3*	*MDM1*	*DAAM1*	*KALRN*	*PCP2*
MMRN1	*RGS3*	*STON2*	*NLRC5*	*MESDC1*	*ESAM*
TSPAN9	*FAM212B*	*KCTD10*	*FAM63A*	*SNORA40*	*KDR*
GNAZ	*LRP12*	*RAP1B*	*IFIT2*	*SEC14L1*	*TMEM40*
MYLK-AS1	*ARHGAP21*	*LIPH*	*SERPINB9*	*NFATC1*	*MX1*
PDE5A	*SRC*	*TAP1*	*LAT*	*RNU5A-1*	*CLEC1B*
IFI6	*PF4*	*DAPP1*	*PTGS1*	*TLN1*	*C10orf10*
GP9	*RGS18*	*RAP2B*	*CXCL8*	*TRIM10*	*F2R*
TREML1	*CXCL2*	*TBXAS1*	*BEX3*	*CD14*	*AQP10*
IFIT1	*MIR1248*	*C12orf76*	*PGRMC1*	*C1orf198*	*SH3BP5*
ASAP2	*ARHGAP6*	*FCGR3A*	*PARVB*	*PRR29*	*XYLT2*
SEPT5	*TTYH3*	*DAB2*	*FN1*	*PRKCB*	*GRK5*
NXF3	*DCK*	*ARL15*	*F13A1*	*NLK*	*P2RY1*
LOC100288069	*SAV1*	*TPM4*	*FOS*	*PLEK*	

Table S3 Network of potential cell-cell interactions between immune MKs and hematopoietic immune cells.

Ligands in immune MKs	Receptors in immune cells	Interactor hematopoietic immune cells	Odds ratio	Adjusted P-value
Anxa1	*Dysf*	Neutrophil		
Anxa1	*Fpr1*	Neutrophil		
Anxa1	*Fpr2*	Neutrophil		
App	*Fpr2*	Neutrophil		
App	*Gpc1*	Neutrophil		
App	*Tnfrsf21*	Neutrophil		
C3	*C5ar2*	Neutrophil		
C3	*Cd46*	Neutrophil		
C3	*Cd81*	Neutrophil		
C3	*Cr1l*	Neutrophil		
C3	*Ifitm7*	Neutrophil	1.66	0.01
C3	*Ifitm2*	Neutrophil		
C3	*Ifitm1*	Neutrophil		
C3	*Ifitm3*	Neutrophil		
C3	*Ifitm6*	Neutrophil		
C3	*Gm49368*	Neutrophil		
C3	*Itgam*	Neutrophil		
C3	*Itgax*	Neutrophil		
C3	*Itgb2*	Neutrophil		
Calm1	*Sell*	Neutrophil		
Calr	*Scarf1*	Neutrophil		

Camp	*Fpr2*	Neutrophil
Camp	*Igf1r*	Neutrophil
Ccl9	*Ccr1*	Neutrophil
Ccl6	*Ccr1*	Neutrophil
Ccl3	*Ccr1*	Neutrophil
Gpi1	*Amfr*	Neutrophil
Hp	*Gm49368*	Neutrophil
Hp	*Itgam*	Neutrophil
Hp	*Itgb2*	Neutrophil
Hsp90b1	*Tlr1*	Neutrophil
Hsp90b1	*Tlr2*	Neutrophil
Hsp90b1	*Tlr4*	Neutrophil
Icam2	*Itgal*	Neutrophil
Icam2	*Gm49368*	Neutrophil
Icam2	*Itgam*	Neutrophil
Icam2	*Itgb2*	Neutrophil
Il16	*Kcnj15*	Neutrophil
Il1rn	*Il1r2*	Neutrophil
Mmp9	*Cd44*	Neutrophil
Mmp9	*Gm49368*	Neutrophil
Mmp9	*Itgam*	Neutrophil
Mmp9	*Itgb2*	Neutrophil
Rps19	*C5ar1*	Neutrophil
S100a8	*Tlr4*	Neutrophil

Selplg	*Gm49368*	Neutrophil		
Selplg	*Itgam*	Neutrophil		
Selplg	*Itgb2*	Neutrophil		
Selplg	*Sell*	Neutrophil		
Tnf	*Ltbr*	Neutrophil		
Tnf	*Tnfrsf1a*	Neutrophil		
Tnf	*Tnfrsf1b*	Neutrophil		
Tnf	*Tnfrsf21*	Neutrophil		
Vim	*Cd44*	Neutrophil		
App	*Cd74*	Monocyte		
App	*Lrp1*	Monocyte		
App	*Ncstn*	Monocyte		
App	*Tnfrsf21*	Monocyte		
Arf1	*Insr*	Monocyte		
C3	*C3ar1*	Monocyte		
C3	*Ifitm7*	Monocyte		
C3	*Ifitm2*	Monocyte	1.19	0.23
C3	*Ifitm1*	Monocyte		
C3	*Ifitm3*	Monocyte		
C3	*Ifitm6*	Monocyte		
C3	*Itgam*	Monocyte		
C3	*Itgax*	Monocyte		
C3	*Itgb2*	Monocyte		
C3	*Lrp1*	Monocyte		

Calm1	*Fas*	Monocyte
Calm1	*Insr*	Monocyte
Calm1	*Kcnq1*	Monocyte
Calr	*Lrp1*	Monocyte
Camp	*P2rx7*	Monocyte
Ccl9	*Ccr1*	Monocyte
Ccl9	*Ccr1l1*	Monocyte
Ccl6	*Ccr1*	Monocyte
Ccl6	*Ccr1l1*	Monocyte
Ccl3	*Ccr1*	Monocyte
Ccl3	*Ccr1l1*	Monocyte
Cd14	*Itga4*	Monocyte
Cd14	*Itgb1*	Monocyte
Gpi1	*Amfr*	Monocyte
Hdc	*Hrh2*	Monocyte
Hp	*Asgr1*	Monocyte
Hp	*Itgam*	Monocyte
Hp	*Itgb2*	Monocyte
Hsp90b1	*Asgr1*	Monocyte
Hsp90b1	*Lrp1*	Monocyte
Hsp90b1	*Tlr2*	Monocyte
Hsp90b1	*Tlr4*	Monocyte
Icam2	*Itgal*	Monocyte
Icam2	*Itgam*	Monocyte

Icam2	*Itgb2*	Monocyte
Il16	*Cd4*	Monocyte
Inhba	*Acvr1b*	Monocyte
Inhba	*Eng*	Monocyte
Ltf	*Lrp1*	Monocyte
Ltf	*Tfrc*	Monocyte
Mmp9	*Cd44*	Monocyte
Mmp9	*Itgam*	Monocyte
Mmp9	*Itgb2*	Monocyte
Mmp9	*Lrp1*	Monocyte
Nampt	*Insr*	Monocyte
Nucb2	*Erap1*	Monocyte
Osm	*Il6st*	Monocyte
Rps19	*C5ar1*	Monocyte
S100a8	*Tlr4*	Monocyte
Selplg	*Itgam*	Monocyte
Selplg	*Itgb2*	Monocyte
Sema4a	*Plxnd1*	Monocyte
Tnf	*Ltbr*	Monocyte
Tnf	*Tnfrsf1a*	Monocyte
Tnf	*Tnfrsf1b*	Monocyte
Tnf	*Tnfrsf21*	Monocyte
Vim	*Cd44*	Monocyte
App	*Cav1*	B cell

App	*Cd74*	B cell		
Bst1	*Cav1*	B cell		
C3	*Cd19*	B cell		
C3	*Cd81*	B cell		
Calm1	*Ptpra*	B cell	0.75	0.82
Il16	*Kcna3*	B cell		
Ltf	*Tfrc*	B cell		
Ncam1	*Ptpra*	B cell		
Nucb2	*Erap1*	B cell		
App	*Cd74*	Macrophage		
C3	*Ifitm2*	Macrophage		
C3	*Ifitm3*	Macrophage		
C3	*Itgax*	Macrophage		
Calm1	*Ptpra*	Macrophage		
Calm1	*Sell*	Macrophage		
Camp	*Igf1r*	Macrophage		
Camp	*P2rx7*	Macrophage	0.76	0.87
Ccl3	*Ccr5*	Macrophage		
Cd14	*Itga4*	Macrophage		
Cd14	*Itgb1*	Macrophage		
Hsp90b1	*Tlr7*	Macrophage		
Hsp90b1	*Tlr9*	Macrophage		
Icam2	*Itgal*	Macrophage		
Igfbp4	*Lrp6*	Macrophage		

Il16	*Ccr5*	Macrophage		
Il16	*Cd4*	Macrophage		
Ltf	*Tfrc*	Macrophage		
Mmp9	*Cd44*	Macrophage		
Ncam1	*Ptpra*	Macrophage		
Orm1	*Ccr5*	Macrophage		
Osm	*Lifr*	Macrophage		
Selplg	*Sell*	Macrophage		
Vim	*Cd44*	Macrophage		
Anxa1	*Dysf*	NK cell		
App	*Gpc1*	NK cell		
C3	*Cr1l*	NK cell		
C3	*Gm49368*	NK cell		
C3	*Itgam*	NK cell		
C3	*Itgax*	NK cell		
C3	*Itgb2*	NK cell		
Calm1	*Sell*	NK cell	0.85	0.78
Ccl3	*Ccr5*	NK cell		
Cd14	*Itga4*	NK cell		
Cd14	*Itgb1*	NK cell		
Hp	*Gm49368*	NK cell		
Hp	*Itgam*	NK cell		
Hp	*Itgb2*	NK cell		
Icam2	*Itgal*	NK cell		

Icam2	*Gm49368*	NK cell		
Icam2	*Itgam*	NK cell		
Icam2	*Itgb2*	NK cell		
Il16	*Ccr5*	NK cell		
Mmp9	*Gm49368*	NK cell		
Mmp9	*Itgam*	NK cell		
Mmp9	*Itgb2*	NK cell		
Nucb2	*Erap1*	NK cell		
Orm1	*Ccr5*	NK cell		
Selplg	*Gm49368*	NK cell		
Selplg	*Itgam*	NK cell		
Selplg	*Itgb2*	NK cell		
Selplg	*Sell*	NK cell		
Tnf	*Tnfrsf1b*	NK cell		
Tnf	*Traf2*	NK cell		
C3	*Ifitm7*	T cell		
C3	*Ifitm2*	T cell		
C3	*Ifitm1*	T cell		
C3	*Ifitm3*	T cell		
C3	*Ifitm6*	T cell	0.59	0.98
C3	*Itgb2*	T cell		
Calm1	*Kcnn4*	T cell		
Calr	*Itgav*	T cell		
Cd14	*Itga4*	T cell		

Cd14	*Itgb1*	T cell
Hp	*Itgb2*	T cell
Icam2	*Itgal*	T cell
Icam2	*Itgb2*	T cell
Il16	*Kcna3*	T cell
Mmp9	*Itgb2*	T cell
Nucb2	*Erap1*	T cell
Selplg	*Itgb2*	T cell
Tnf	*Tnfrsf1b*	T cell

Table S4 Primers of immune receptors and mediators.

Gene	Forward (5'-3') primer	Reverse (5'-3') primer
Gapdh	TGAAGGTCGGTGTGAACGGATT	CTCGCTCCTGGAAGATGGTGAT
C5ar1	ACCGCCTGTATAGTCCTGC	GGTCGGCACTAATGGTAGCC
Fpr1	CCATTTGGTTGGTTCATGTGC	CTTCTTGGCTAGGCTCACAGT
Tlr4	CCGCTCTGGCATCATCTTCATTG	CTCTGCTGTTTGCTCAGGATTCG
Tlr2	CCCTTCTCCTGTTGATCTTGCT	CGCCCACATCATTCTCAGGTA
Ifngr1	CGAAGCAGCAGAACAGGAAGAAC	TGATAGGCGGTGAGGCTACAAG
S100a8	TGAGTGTCCTCAGTTTGTGCAG	TGCCACACCCACTTTTATCACC
Camp	GGCTGTGGCGGTCACTATC	GTCTAGGGACTGCTGGTTGAA
Hck	TCGTTGTCTGTTCGAGACTTTG	TCTTGTAGTGGAGCACGAGTT
Grn	TTATGGTTGATGGTTCGTGGG	GGGGACAGCAATGCACTCT
Lcn2	TGGCCCTGAGTGTCATGTG	CTCTTGTAGCTCATAGATGGTGC
Ccl3	ACCATGACACTCTGCAACCA	GATGAATTGGCGTGGAATCT

Table S5 Detail information of antibodies.

Antibody	Company	CAT.NO.	Dilution
Human CD41a APC	BD bioscience	Cat#559777	1：100
Human CD41a FITC	BD bioscience	Cat#555466	1：100
Human CD42b FITC	BD bioscience	Cat#555472	1：100
Human CD42b APC	BD bioscience	Cat#551061	1：100
Human CD42b PE	BD bioscience	Cat#555473	1：100
Human CD148 PE	Biolegend	Cat#328708	1：100
Human CD48 FITC	BD bioscience	Cat#555759	1：100
Human CD48 BV421	BD bioscience	Cat#562718	1：100
Mouse CD41 PE	Biolegend	Cat#133906	1：100
Mouse CD41 FITC	BD bioscience	Cat#553848	1：100
Mouse CD41 pacific blue	BD bioscience	Cat#133932	1：100
Mouse CD41 APC	Biolegend	Cat#133914	1：100
Mouse CD42d APC	BioLegend	Cat#148506	1：100
Mouse CD42d PerCP-cy5.5	BioLegend	Cat#148508	1：100
Mouse CD148 PE	BD bioscience	Cat#565747	1：100
Mouse CD48 FITC	Biolegend	Cat#103404	1：100
Mouse CD48 Pacific blue	Biolegend	Cat#103418	1：100
Mouse C5AR1 APC	Biolegend	Cat#135807	1：100
Mouse TLR4 PE-Cy7	Biolegend	Cat#145408	1：100
Mouse Ly6G PE	BD bioscience	Cat#561104	1：200
Mouse S100A8	Proteinteck	Cat#15792-1-AP	1：100
Mouse CAMP	Proteinteck	Cat#12009-1-AP	1：100

Mouse HCK	Proteinteck	Cat#11600-1-AP	1：100
Mouse GRN	Proteinteck	Cat#18410-1-AP	1：100
Mouse LCN2	Proteinteck	Cat#26991-1-AP	1：100
Mouse CCL3	Bioss	Cat#bs-1045R	1：100
Mouse CD48	Santacruze	Cat#sc-8397	1：100
Mous Endomucin	Santacruze	Cat#sc-65495	1：200
Hoechst 33342	Solarbio	Cat#C0031	1：1000

Supplemental Experimental Section

Library construction and single cell RNA-Seq

For human MKs isolated in vivo, the single $CD41a^{+}CD42b^{+}$ cells were immediately transferred into lysis buffer by manual picking. The single-cell RNA-seq library preparation and sequencing were performed based on the modified Smart-Seq2 protocol.[58] Briefly, the samples were vortexed and incubated at 72℃ for 3 min before reverse transcription PCR with template switch oligo (TSO) primer. Subsequently, the cDNAs were amplified by 14 cycles of PCR with 3'P2 primer and IS primer. Samples were pooled and purified using Agencourt AMPure XP beads (Beckman) and 4 cycles of PCR were performed to introduce biotin-modified index sequence. Then, cDNAs were fragmented to around 300 bp using Covaris S2 (Covaris) and enriched by Dynabeads MyOneTM Streptavidin C1 beads (Thermo Fisher). Libraries were constructed using KAPA Hyper Prep Kit (Kapa Biosystems) and sequenced on Illumina Hiseq X ten platform. For mouse MKs isolated in vivo, the single $CD41^{+}CD42d^{+}$ cells were treated with the same Smart-Seq2 protocol and sequenced on NovaSeq 6000 platform.

For human MK differentiated in vitro, total cells on day 0, 4, 8 and 12 of MK differentiation from BM-$CD34^{+}$ HSPCs were collected and resuspended in PBS buffer with 1% BSA at a density of 1×10^{6}/mL. The cell viability was more than 95% as revealed by trypan blue exclusion. Cells were lyzed, and barcoded oligonucleotides were mixed with cDNA. Single-cell RNA-seq libraries were prepared using 10X_Single_cell_RNA-seq 3 library Kit V2, and sequencing was performed on Illumina_NovaSeq_5000 with pair end 150bp (PE150).

Data preprocessing and quality control

For Smart-Seq2, adapters were cleaned with Cutadapt before the raw data

were split to independent single cells by the use of specific barcodes. The alignment of human sequencing data was completed with GRCh38 human reference genome using STAR. The same pipeline aligned with Genome mm10 mouse reference genome was applied to mouse sequencing data. To filter low-quality cells, the initial quality control was performed by using Seurat (version 3.1.5) implemented in R (version 4.0.0) and cells with fewer than 200 genes or more than 10,000 genes were discarded. To explore the potential effect of nCount_RNA on cell clustering, we regressed out the nCount_RNA by using ScaleData function in Seurat and found that regressing out nCount_RNA has no significant effect on clustering and cell types. Because MK development is an energy-requiring process and higher number of mitochondria are needed to complete the MK maturation faster,[59-60] the adult mature MKs may have high mitochondrial expression. Also, MKs with higher mitochondrial gene expression exhibited high counts of unique molecular identifier (UMI) and high expression of housekeeping genes. In order to retain as complete cell information as possible, we chose the lower quartiles of the mitochondrial expression as the filtering threshold in each data (the cells with >70% in human MKs and >40% in mouse MKs were excluded, respectively). Genes that were expressed in fewer than 3 cells were also excluded. In eight human samples, 860 MKs in total passed the quality control, with 4,595 genes and 427,814 counts detected per cell on average. For mouse MKs, 851 cells (457 MKs from four control mice and 394 MKs from seven infected mice) passed the quality control, with 5,916 genes and 1,165,517 counts detected per cell on average.

The raw data from 10 × genomics platform were aligned and quantified using the Cell Ranger count pipeline 2.1.0 with GRCh38 human reference genome using STAR (http://www.10xgenomics.com). To filter low-quality cells, the initial quality control was performed to discard cells with fewer than 200 genes or more than 5,000 genes. The lower quartiles of the mitochondrial

expression were also chosen as the filtering threshold to exclude those cells with >10% of mitochondrial gene expression. We also performed doublet-removal for each 10 × Genomics dataset using Python-based Scrublet software (version 0.2.1) with the recommended parameters.[61] Also, genes expressed in fewer than 3 cells were excluded. In brief, out of the total 16,159 cells sequenced in all timepoints, 15,780 cells passed the quality control, with 3,064 genes and 16,726 counts detected per cell on average.

Cell clustering and dimensionality reduction

After quality control was completed, the Seurat package (version 3.1.5) implemented in R (version 4.0.0) was used to perform downstream analysis and visualization of all the data. Specifically, the sequencing data were normalized by default function 'NormalizeData' in Seurat using logarithmic transformation, and the scale factor was set to 10,000. Top 2,000 high variable genes were calculated by 'FindVariableFeatures' in Smart-Seq2 datasets and 2,500 high variable genes in 10 × genomics datasets, respectively. Then scaling was applied to eliminate the impact of mitochondrial gene variations. Afterwards, principal component analysis (PCA) was conducted based on the expression matrix of RNA-slot features and the top 20 significant PCs for human MKs, top 15 significant PCs for mouse MKs and human in vitro cells were selected to perform dimensionality reduction and clustering. Cells were projected into a two-dimension space using Uniform Manifold Approximation and Projection (UMAP) with default parameters and cell clusters were identified using the 'FindClusters' and 'RunUMAP' function from Seurat. Finally, five clusters for human Smart-Seq2 datasets (resolution = 0.5), five clusters for mouse Smart-Seq2 datesets (resolution = 0.15) and twelve clusters for human 10 × Genomics dataset were identified for subsequent analysis.

Identification and analysis of differentially expressed genes

The differentially expressed genes between different clusters were identified

using the ‘FindAllMarkers’ function implemented in Seurat with Wilcoxon rank sum test. Significant differentially expressed genes were selected by P value adjusted using bonferroni correction (< 0.05). The list of surface markers used in this study was obtained from Human Cell Differentiation Molecules (http://www.hcdm.org).

The gene profiles with MK differentiation

The signature genes dynamically expressed along MK differentiation (from MEP, via MKP to MK) were calculated by using function AverageExpression in Seurat and then visualized on the distinct two developmental hierarchies.

Bioinformatic inference of cell-cell interactions

Marker genes of hematopoietic immune cells in the BM were downloaded from the published data.[32,62-63] To aggregate immune cell genes into a consensus set, the union of all identified marker genes was taken. The marker genes of each mouse MK subset were obtained from the single cell RNA-seq data as described in the previous section. Receptor-ligand pairs were downloaded from CellTalker.[39] On the basis of these lists of marker genes and receptor-ligand pairs, we inferred potential cell-cell interactions for all pairs of each MK subpopulation and immune cells. First, we separately counted the matching interaction pairs of each MK subset and hematopoietic immune cell as ligands and receptors. The corrplot R package was used for visualization. Second, we tested whether the interaction between immune MKs and immune cells was greater than that of other MKs. Fisher’s exact test was used to obtain P values and odds ratios, as implemented by using the function ‘fisher.test’ in R. Finally, enriched pairs of immune MKs and immune cell types were connected by solid (adjusted P value < 0.05) or dashed (adjusted P value > 0.05) lines to generate a graph of cell-cell interactions.

The immune-biased megakaryopoiesis in vitro

Umbilical cord blood units were obtained from healthy full-term neonates with informed consent from the parents and approved by the ethics committee of the Institute of Hematology and Blood Diseases Hospital, Chinese Academy of Medical Sciences. Megakaryocytic differentiation of $CD34^+$ cells were performed as previously described.[64] For induction of immune-biased megakaryopoiesis, LPS (0, 0.1, 1, 10, 20 μg/mL) or IFN-γ (0, 10, 20, 100, 500 ng/mL) was added into the culture medium on the 10th day of differentiation and stimulated 2 days before MK detection.

Mouse acute inflammation model

Eight-to-Ten-week-old C57/BL6 female mice were intraperitoneally injected with 1×10^7 colony forming unit of heat-inactivated E. coli suspended in 300 μL 0.9% NaCl to induce acute inflammatory response. 6, 12, 24, 30, 36, 48 and 72 h post injection, mice were sacrificed via cervical dislocation. Platelet count in peripheral blood post E.coli infection was determined by using an automated blood cell analyzer (BC-5000 Vet, Mindray). Cells were collected from the BM and spleen for flow cytometric analysis.

May-Grunwald-Giemsa staining

Cells were collected, suspended in 40 μL PBS, and subsequently cytospun onto polylysine-coated slides using cytospin (Thermo Shandon). The dried slides were stained with May-Grünwald-Giemsa (MGG) staining solution (Beyotime) for 15 min according to the manufacturer’s instructions. Images were captured using a Nikon microscope.

Sample preparation and flow cytometry

To determine the proportion of defined populations in aforementioned differentiation culture conditions, the differentiated cells were collected in 0.01% BSA and labeled with fluorescein-conjugated antibodies for 30 min in the dark. For cells isolated in vivo, cells from human or mouse BM were

flushed out gently with DPBE (2% FBS and 0.4% 0.5M EDTA in DPBS) buffer. Spleen cells were grinded and suspended with DPBE buffer for detection. All cells were incubated with antibodies at 4℃ for 30 min in the dark. For the detection of intracellular soluble proteins, cells were fixed with 4% PFA for 15 min and permeabilized with 0.1% Triton X-100 for 15 min before staining. Flow cytometry analysis was performed using a FACS CantoII flow cytometer (BD Biosciences). Detailed information of the antibodies is listed in Table S5.

Single-cell qPCR

The single-cell qPCR (quantitative polymerase chain reaction) analysis was performed based on a modified protocol.[65] Briefly, individual primer sets (Table S4) were pooled to a final concentration of 0.1 μM for each primer. The $CD148^{+}CD48^{-}$ and $CD148^{+}CD48^{+}$ MKs sorted from the bone marrow of immune-challenged mice were manually picked into 8-Tube Strip (0.2 mL) loaded with 5 μL RT-PCR master mix (2.5 μL 2× Reaction Mix, Vazyme Single Cell Sequence Specific Amplification Kit; 0.5 μL primer pool; 0.1 μL RT/Taq Enzyme, Vazyme Single Cell Sequence Specific Amplification Kit; 1.9 μL nuclease-free water) in each well. Sorted cells were immediately frozen at -80℃ for 5 min. After brief centrifugation at 4℃, cell lysis and sequence-specific reverse transcription were performed at 50℃ for 60 min. Then reverse transcriptase inactivation and Taq polymerase activation were achieved via heating to 95℃ for 3 min. Subsequently, cDNA underwent 20 cycles of sequence-specific amplification via denaturing at 95℃ for 15 s, annealing and elongation at 60℃ for 15 min. After pre-amplification, PCR tubes were stored at -80℃ to avoid evaporation. Pre-amplified products were diluted 5-fold prior to analysis. Real-time PCR analysis was performed using QuantiTech SYBR Green PCR kit (Qiagen, Hilden, German).

Cryosectioning of mice bone

Mouse femurs were fixed in 4% PFA, decalcified in 0.5M EDTA, and

dehydrated in 30% sucrose in 4℃ prior to embedding in EBM and freezing. Frozen femurs were then cut into 40 μm sections using cryostat (Leica) before immunofluorescence studies.

Immunofluorescence

Cultured cells, smears and frozen sections from humans and mice were fixed with 4% PFA in PBS for 30 min, permeabilized with 0.1% Triton X-100 in PBS for 30 min, and blocked with 1-5% BSA in PBS for 1 h. Cells were then incubated with different antibodies at 4 °C overnight. The nuclei were stained with Hoechst 33342 for 10 min before experimentation. Images were acquired using spinning disk confocal microscope (UltraVIEW VOX) and analyzed with Volocity. Detailed information and dilutions for the antibodies are listed in Table S6.

Cellular localization analysis of MKs

Images of femur frozen sections, collected from the spinning disk confocal microscope (Dragonfly 200), were used to produce spatial maps of MKs in the marrow. Background staining was removed to accurately represent MKs and blood vessels. $CD48^-$ MKs and $CD48^+$ MKs were selected from the defined region, and the shortest Euclidean distance was calculated for each MKs to blood vessels. The distance distributions were analyzed with Imaris software (Bitplane) and ImageJ.

Supplemental references

[59] I.C. Macaulay, J.N. Thon, M.R. Tijssen, B.M. Steele, B.T. MacDonald, G. Meade, P. Burns, A. Rendon, V. Salunkhe, R.P. Murphy, C. Bennett, N.A. Watkins, X. He, D.J. Fitzgerald, J.E. Jr. Italiano, P.B. Maguire, *Blood* **2013**, 121, 188.

[60] R.B. Undi, U. Gutti, R.K. Gutti, *J. Trace. Elem. Med. Biol.* **2017**, 39, 193.

[61] S.L. Wolock, R. Lopez, A.M. Klein, *Cell Syst*. **2019**, 8, 281.

[62] X. Han, R. Wang, Y. Zhou, L. Fei, H. Sun, S. Lai, A. Saadatpour, Z. Zhou, H. Chen, F. Ye, D. Huang, Y. Xu, W. Huang, M. Jiang, X. Jiang, J. Mao, Y. Chen, C. Lu, J. Xie, Q. Fang, Y. Wang, R. Yue, T. Li, H. Huang, S. H. Orkin, G. C. Yuan, M. Chen, G. Guo, *Cell* **2018**, 172, 1091.

[63] Tabula Muris Consortium, Overall coordination, Logistical coordination, Organ collection and processing, Library preparation and sequencing, Computational data analysis, Cell type annotation, Writing group, Supplemental text writing group, Principal investigators, *Nature* **2018**, 562, 367.

[64] Y. Yang, C. Liu, X. Lei, H. Wang, P. Su, Y. Ru, X. Ruan, E. Duan, S. Feng, M. Han, Y. Xu, L. Shi, E. Jiang, J.Zhou, *Stem Cell. Transl. Med*. **2016**, 5, 175.

[65] G. Guo, S. Luc, E. Marco, T.W. Lin, C. Peng, M.A. Kerenyi, S. Beyaz, W. Kim, J. Xu, P.P. Das, T. Neff, K. Zou, G.C. Yuan, S.H. Orkin, *Cell Stem Cell* **2013**, 13,492.

[66] H. Raslova, A. Kauffmann, D. Sekkaï, H. Ripoche, F. Larbret, T. Robert, Le. Roux. D. Tronik, G. Kroemer, N. Debili, P. Dessen, V. Lazar, W. Vainchenker, *Blood* **2007**, 109, 3225.

[67] H. Raslova, L. Roy, C. Vourch, J.P. Le Couedic, O. Brison, D. Metivier, J. Feunteun, G. Kroemer, N. Debili, W. Vainchenker, *Blood* **2003**, 101, 541.

[68] K. Eto, S. Kunishima, *Blood* **2016**, 127, 1234.
